# Glycan recognition by collectin-11 drives SARS-CoV-2 infectivity and membrane injury of respiratory epithelial cells

**DOI:** 10.1073/pnas.2521209122

**Published:** 2025-10-24

**Authors:** Anastasia Polycarpou, Tara Wagner-Gamble, Roseanna Greenlaw, Lauren O’Neill, Varsha Kanabar, Alanoud Alrehaili, Yusun Jeon, Jonathan Baker, Mona Bafadhel, Hataf Khan, Michael H. Malim, Marco Romano, Conrad A. Farrar, Dorota Smolarek, Rocio Martinez-Nunez, Katie J. Doores, Russell Wallis, Linda S. Klavinskis, Steven H. Sacks

**Affiliations:** ^a^Peter Gorer Department of Immunobiology, School of Immunology and Microbial Sciences, King’s College, London SE1 9RT, United Kingdom; ^b^Department of Infectious Diseases, School of Immunology and Microbial Sciences, King’s College London, London SE1 9RT, United Kingdom; ^c^Department of Respiratory Sciences, University of Leicester, Leicester LE1 9HN, United Kingdom; ^d^King’s Centre for Lung Health, School of Immunology and Microbial Sciences, King’s College London, London SE1 9RT, United Kingdom

**Keywords:** collectin-11, glycans, complement proteins, SARS-CoV-2

## Abstract

SARS-CoV-2 respiratory-tract infection affects both vaccinated and unvaccinated persons suggesting factors besides adaptive immunity are operative. We investigated the role of collectin-11 (CL-11), an epithelial-secreted carbohydrate-binding lectin that drives innate immunity and eliminates pathogens by complement activation. SARS-CoV-2, despite binding CL-11 to activate complement, was resistant to lysis. Remarkably, opsonization by CL-11 enhanced virus production by infected respiratory epithelial cells independently of complement. Furthermore, infected cells expressing SARS-CoV-2 spike protein displayed enhanced vulnerability to CL-11 binding and membrane attack by complement. The mechanism of enhanced infectivity was ablated in the presence of L-fucose, which occupied the extended carbohydrate-binding cleft of CL-11 in a crystallographic analysis of complexes between L-fucose and CL-11. Our study suggests pathogenicity of SARS-CoV-2 is related to complement-resistance together with enhanced infectivity and injury of respiratory epithelial cells mediated by locally released CL-11.

## Significance

SARS-CoV-2 infection of the respiratory tract continues to be a health risk even among immunized individuals suggesting that localized factors could maintain viral infection and transmission. Here, we show that although the locally produced innate immune-surveillance molecule collectin-11 (CL-11) is bound by SARS-CoV-2 to trigger complement activation, the virus is resistant to complement lysis offering a means of immune escape. Moreover, we reveal CL-11 binding enhances SARS-CoV-2 infectivity of respiratory epithelial cells (RECs) by a complement-independent mechanism affording highly transmissible variants of SARS-CoV-2 a survival advantage. Additionally, SARS-CoV-2-infected RECs are vulnerable to membrane injury by self-generated CL-11 and complement. These insights provide a strong rationale for CL-11 blockade to reduce SARS-CoV-2 infection and injury of the respiratory tract.

Vaccination against SARS-CoV-2 markedly reduces the rate of severe infection and hospitalization but it does not guarantee against infection of the respiratory tract (1, 2). Indeed, vaccinees infected with SARS-CoV-2 have similar upper respiratory viral loads as unvaccinated individuals who acquire the infection, whether they are symptomatic or not (3, 4). It is possible that antigen-specific immunity, while protecting against systemic infection, has less control over the peripheral compartment including the respiratory mucosa—a factor that could contribute to the human reservoir of SARS-CoV-2 and onward transmission. Better understanding of the role of innate immunity is required particularly where virulent pathogens are adapted for immune evasion (5–8). In fact, the association between Long COVID and complement activation suggests that incomplete resolution of infection or inflammation are at play (9, 10).

Of particular interest is the pattern-recognition molecule collectin-11 (CL-11), whose structure resembles the better known C-type lectin mannose-binding lectin (MBL), but which differs because it is produced ubiquitously at epithelial surfaces including the respiratory tract (11, 12) whereas MBL is exclusively expressed in hepatocytes and the circulation. Upon contact with pathogen-associated (11) or damage-associated (13, 14) glycan ligands, CL-11 interacts with other locally produced complement components (15) resulting in lectin complement pathway activation. The fundamental subunit of CL-11 includes a carbohydrate-recognition domain (CRD) and a collagen-like domain (CLD). These subunits combine to form multivalent trimers and multiples of trimers with potential for cross-linking carbohydrate ligands, typically fucosylated glycans and high-mannose oligosaccharides (11, 16). MBL-associated serine proteases (MASPs 1-3) (17, 18) complexed with the CLD of CL-11 mediate the cleavage of C3 and then C5, leading to the generation of complement effectors that participate in opsonization (C3b), inflammation (C3a, C5a), and lysis (C5b-9) (16).

Clinicopathological studies have highlighted the association between severity of SARS-CoV-2 infection (COVID-19) and components of lectin complement-pathway activation e.g., MASP-2 and C3b (19–21) detected in tissues alongside viral antigen (19, 21) and inflammation (21–23). In addition, a genetic-association study has linked CL-11 and complement to severity of COVID-19 (24), implying a causal relationship between glycan recognition and the ensuing innate immune response. Crucially, a model of the structure of SARS-CoV-2 spike protein—instrumental for receptor binding and the membrane-fusion process of cell entry—shows the position of multiple glycosylation sites for recognition by CL-11 (25). Taken together, these clinical, genetic, and structural observations suggest that the role of CL-11 in monitoring respiratory tract pathogens has been underestimated as a risk factor in SARS-CoV-2 infection. Here, we present functional data for how CL-11 interacts with both the virus and the host cell to impact infectivity and cell injury, and we explore the potential for therapeutic manipulation of CL-11 interacting with glycan ligand.

## Results

### SARS-CoV-2 Binds CL-11 by Its Carbohydrate Recognition Site.

**Fig. 1.**
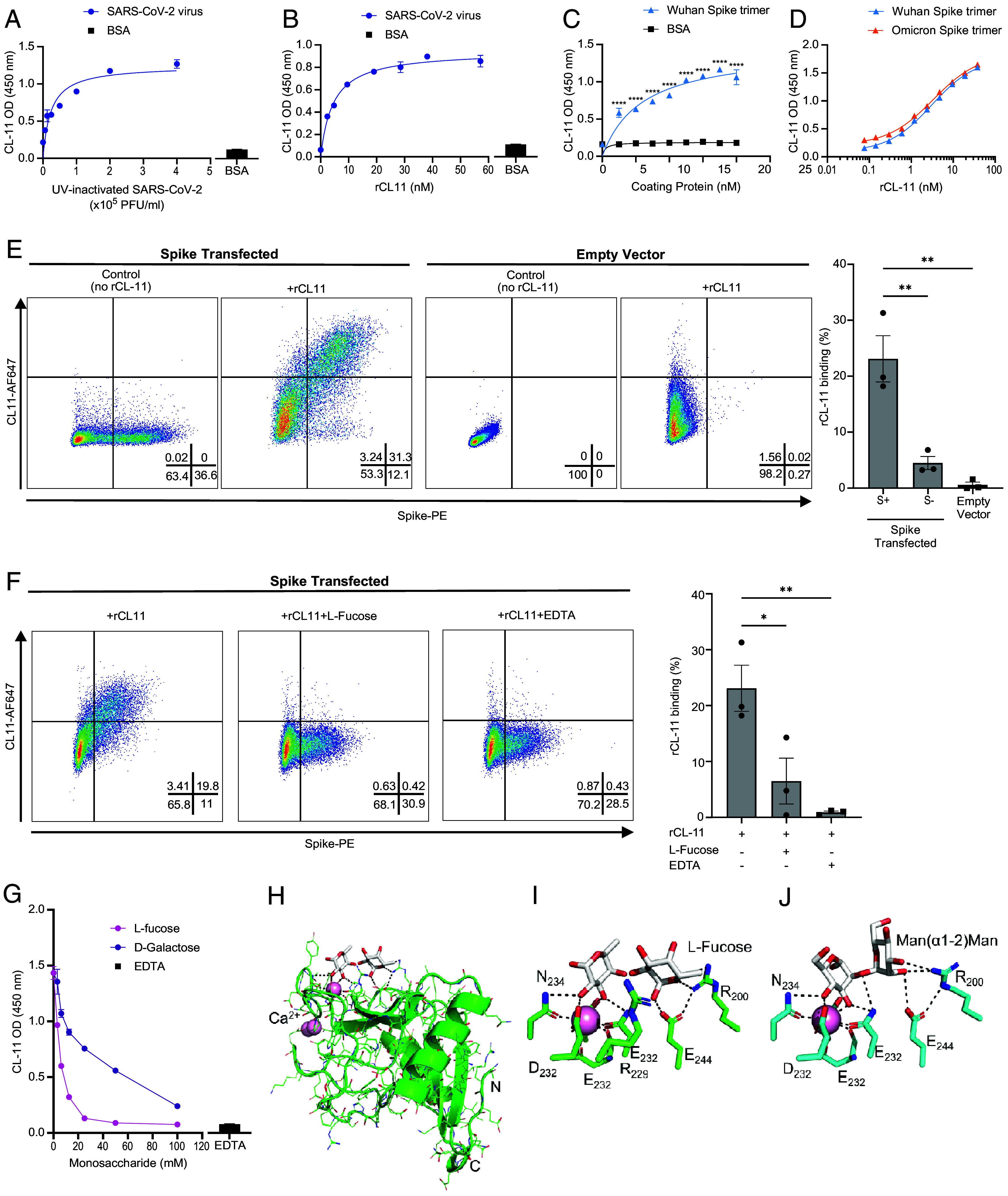
SARS-CoV-2 interaction with the carbohydrate-binding domain of CL-11 is blocked by L-fucose. (*A*–*D*) Measurement of human rCL-11 bound to immobilized UV-inactivated SARS-CoV-2 (*A* and *B*) or immobilized spike protein or BSA (*C* and *D*) detected by ELISA with specific antibody to human CL-11. (*A*), rCl-11 (3 µg/mL) added to titration of SARS-CoV-2 virions (or BSA). (*B*), titration of rCL-11 added to SARS-CoV-2 virions (2 × 10^5^ PFU/mL equivalent) or BSA. (*C*), rCl-11 (3 µg/mL) added to Wuhan spike trimer or BSA over a concentration range. (*D*), titration of rCL-11 added to Wuhan or Omicron spike trimer (2 µg/mL). (*E* and *F*), Representative contour plots of rCL-11 bound to cell-surface expressed spike protein analyzed by flow cytometry. (*E*), *Left*-two panels, spike-transfected HEK293T cells, and *Right*-two panels, empty-vector transfected HEK293T cells, treated with either PBS (control) or rCL-11 (25 µg/mL) prior to staining for cell surface CL-11 and spike protein. Bar graph summarizes percent of cells with bound rCL-11 gated for cell surface spike protein (±) or within the control (empty vector)-transfected cell population. (*F*), rCL-11 pretreated with PBS, L-fucose (10 mM), or EDTA (10 mM) prior to incubation with spike transfected HEK293T cells. (*G*) ELISA data show sugar specificity and Ca^2+^ binding dependence of CL-11 bound to immobilized Wuhan spike trimer (2 µg/mL), where rCL-11 (3 µg/mL) had been pretreated with L-fucose or D-galactose (0 to 100 mM) to block carbohydrate recognition of CL-11, or with EDTA (10 mM). (*H*) Structure of the rCL-11 CRD (green) bound to fucose (white). (*I*) Two fucose residues bind the rCL-11 CRD, one in the primary site forming contacts with Ca^2+^ (pink) and its coordinating amino acid residues. The second occupies an adjacent subsite forming hydrogen bonds with the side chains of Glu244 and Arg200 and packing against the side chain of Arg229. (*J*) CL-11 (blue) binds to high-mannose oligosaccharides, a natural ligand, via its terminal Man(α1-2) Man residues (PDB: 4YMD). Fucose blocks both binding subsites in the CRD. (*A*–*G*) Data representative of at least three independent experiments, error bars show SEM, *P < 0.05; **P < 0.01; ****P < 0.0001; [two-way ANOVA with Šidák’s correction for (*C*), one-way ANOVA with Tukey’s multiple comparison for (*E*, *F*)].

We first verified that our recombinant CL-11 (rCL-11) protein interacts with SARS-CoV-2 virions and spike protein, as predicated from a model of the structure of the SARS-CoV-2 spike protein, which revealed multiple glycan signatures that can be recognized by CL-11 (25). ELISA with immobilized UV-inactivated SARS-CoV-2 virions or recombinant spike protein confirmed binding of rCL-11 in proportion to the amount of virus or spike trimer present, while control binding with a nonglycosylated protein (BSA) was negligible (Fig. 1 *A*–*C*). Importantly, the ancestral Wuhan spike protein and the later Omicron BA.1 variant spike protein displayed equivalent binding affinity for rCL-11 [EC_50_ values of 7.6 (±4.2) × 10^−9^ M and 6.0 (±2.6) × 10^−9^ M respectively], Fig. 1*D*. Comparison of our in-house rCL-11 with a commercial rCL-11 generated in a wheat germ expression system, to address differences in our spike binding data with those reported in a previous study (26), revealed strong binding by our CHO-expressed rCL-11 yet negligible binding of the wheat germ-derived rCL-11 from the same supplier as the reported study (*SI Appendix*, Fig. S1). This discrepancy between the two recombinant proteins could have been due to differences in protein folding required for functional activity of rCL-11, possibly related to lack of posttranslational modifications such as hydroxylation of proline and lysine residues or disulfide bond formation inherent in the wheat germ system that are important for stability of the CLD (27) and oligomerization of collectins (28).

We next assessed whether membrane-expressed spike protein would also allow CL-11 binding. For this, we incubated spike-transfected HEK293T cells with a fixed amount of rCL-11. Flow cytometry demonstrated significant binding of rCL-11 to cells expressing cell-surface spike compared to transfected cells negative for surface-expressed spike or empty vector (EV) control-transfected cells (Fig. 1*E* and gating strategy *SI Appendix*, Fig. S2). These data provided strong evidence that CL-11 readily interacts with SARS-CoV-2 spike protein when the protein is expressed on cells or immobilized on plastic.

In theory, binding of CL-11 to a target surface could occur either through the CRD of CL-11 or its CLD. It is recognized that binding by the CRD of C-type lectins such as CL-11 has two distinct properties: Ca^2+^-dependent binding to the target substrate; and sugar-inhibitable binding. Using HEK293T-cell-expressed spike trimer or plate-immobilized spike trimer as the binding target we demonstrated rCL-11 binding to be both EDTA and sugar-inhibitable (Fig. 1 *F* and *G*). Treatment with increasing concentrations of L-fucose to saturate the CRD of CL-11 typically inhibited rCL-11 binding to the target by >90%. Likewise, the Ca^2+^ chelator EDTA inhibited the CL-11/spike interaction by a similar amount. Inhibition was less effective with D-galactose control (Fig. 1*H*), which has been shown to have significantly lower binding affinity for CL-11 than L-fucose (16). In addition, we confirmed that the interaction of CL-11 with spike protein was also inhibitable by D-mannose, to a comparable extent as L-fucose (*SI Appendix*, Fig. S2). Collectively these data indicate that interaction between the SARS-CoV-2 spike protein and CL-11 is mediated by the CRD of CL-11, an interaction which is blocked in the presence of high concentrations of L-fucose.

To understand how L-fucose so ably blocks the CRD of CL-11 and thereby prevents CL-11 binding to relevant ligands, e.g., oligomannose- or complex-type glycan ligands that can occur at N-glycosylation sites of the spike protein (25, 29), we examined the crystal structure of complexes formed between the CRD of CL-11 and L-fucose (30; *SI Appendix*, Table S1). The crystal contained two CRD molecules in the asymmetric unit each bound to three Ca^2+^ ions (Fig. 1*H*). Density for fucose was observed in the primary binding site of each CRD. The equatorial 2- and 3-hydroxyl groups of the fucose bound directly to the Ca^2+^ and form polar contacts with Ca^2+^-coordinating residues: Glu232, Asn234, Glu240, and Asn252 (Fig. 1*I*). Unexpectedly, density for a second fucose occurred in the adjacent subsite of one of the CRDs, forming hydrogen bonds with the side chains of Glu244 and Arg200 and packing against the side chain of Arg229. This subsite was inaccessible to ligand in the second CRD because of crystal contacts. These data indicate L-fucose blocks both binding subsites in the CRD, and this provides the structural basis for how L-fucose efficiently blocks carbohydrate-recognition of natural ligands [e.g., high-mannose oligosaccharides on the SARS-CoV-2 spike protein (29) via the terminal Man(α1-2)Man residues (Fig. 1*J*) (31)]. The structures of the two CRDs in the current model (Fig. 1*I*) are like the 6 CRDs observed in PDB:4YMD (Fig. 1*J*), with RMSD values of 0.32-0.37 over 790-811 atoms for one CRD and 0.33-0.38 over 789-826 atoms for the other.

### SARS-CoV-2 Triggers Complement Activation.

**Fig. 2.**
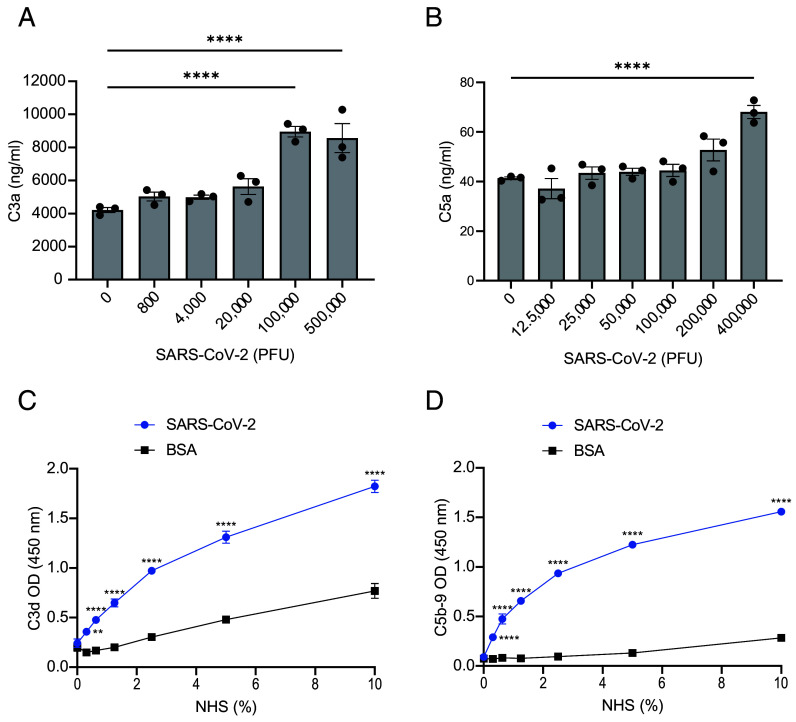
SARS-CoV-2 initiates complement activation. (*A* and *B*) Quantification by ELISA of C3a (*A*) and C5a (*B*) generated in NHS after incubation (1:1 by volume) with live SARS-CoV-2 virions. (*C* and *D*) Quantification by ELISA of C3d and C5b-9 deposited on immobilized UV-inactivated SARS-CoV-2 virions (2 × 10^5^ PFU/mL equivalent) or BSA (as control) after incubation with NHS for 1 h at 37 °C. Deposited C3d (*C*) and C5b-9 (*D*) detected with specific antisera against C3d and C5b-9 followed by enzyme-conjugated anti-species detection antibodies. Data are representative of three (*A*, *C*, and *D*) and two (*B*) independent experiments respectively. Error bars represent the mean ± SEM of triplicate biological samples. **P < 0.01, ***P < 0.001, ****P < 0.0001 [one-way ANOVA with Dunnett’s multiple comparisons against no virus control (*A* and *B*), two-way ANOVA with Šidák’s multiple comparison (*C* and *D*)].

Because rCL-11 at an equivalent concentration to the level in normal human serum (NHS; 0.1 to 0.5 μg/mL; 0.95 to 4.76 nM) was bound by SARS-CoV-2 (Fig. 1 *B* and *D*), we examined how well SARS-CoV-2 could initiate complement activation in NHS (sero-negative for SARS-CoV-2 spike antibody), where other essential complement components are present. In the first approach, we measured the soluble complement-activation products C3a and C5a released into the supernatants after incubating SARS-CoV-2 virions with NHS as the source of complement. The levels of C3a and C5a generated were significantly above those in control samples in the absence of added virus (Fig. 2 *A* and *B*). Basal levels of C3a and C5a detected in these control assays are likely to reflect the presence of these products in NHS (32, 33) or may represent low-level complement activation on the plastic surface of the assay system.

In a second approach, we examined the deposition of C3d and C5b-9 on UV-inactivated SARS-CoV-2 virions immobilized on plastic wells, where C3d is the end metabolite of bound C3b deposited on an activating surface and C5b-9 is MAC formed after cleavage of C5. We detected significant increases of bound C3d and C5b-9, as compared with control measurements where immobilized BSA replaced the virus (Fig. 2 *C* and *D*). The deposition of complement was dose-sensitive over a range of serum dilutions (0 to 10% NHS). These data indicate a low threshold for complement activation by SARS-CoV-2 under the assay conditions.

### SARS-CoV-2 Is Resistant to Complement-Mediated Lysis.

**Fig. 3.**
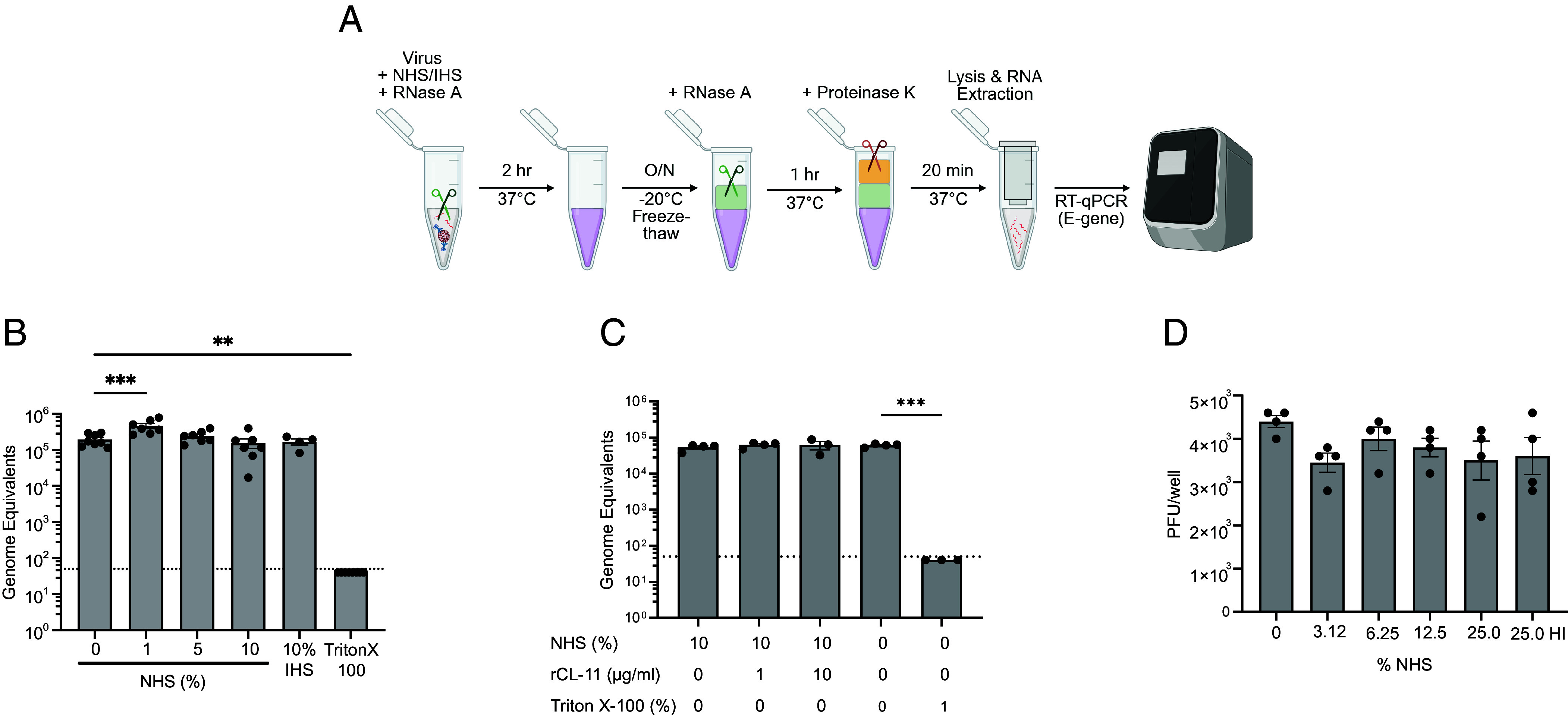
SARS-CoV-2 virions are resistant to complement-mediated lysis. (*A*) Workflow to determine resistance of SARS-CoV-2 to complement by virion lysis assay, using pooled NHS (final concentration 0, 1, 5, and 10%) and IHS (10%) in the presence of SARS-CoV-2 virions and RNase A. Absolute viral RNA quantitation was determined by RT-qPCR and expressed as number of SARS-CoV-2 E genome copies. (*B*) Resistance of SARS-CoV-2 virions to lysis by pooled NHS (final concentration 0, 1, 5, and 10%), (*C*) Resistance of SARS-CoV-2 virions to lysis by 10% NHS in the presence of rCL-11 (1.0 and 10.0 µg/mL final concentration). Triton-X-100 (1% final concentration) included as a control. Data represent the mean ± SEM of at least triplicate biological samples assayed in duplicate by RT-qPCR and are representative of three independent experiments. (*D*) SARS-CoV-2 virus (10^4^ PFU) was preincubated with NHS (0 to 25%) or with 25% HI NHS (as control) for 1 h at 37 °C and then applied to Vero E6 TMPRSS2 cells. Infectivity determined by plaque assay with readouts shown in PFU. Data are pooled from two independent experiments performed with duplicate biological samples. (*B*–*D*) Each group compared to 0% NHS by one-way ANOVA with Dunnett’s multiple comparison test, **P < 0.01 and ***P < 0.001.

Given the pathogenicity of SARS-CoV-2 despite activating innate complement defense (19–21), we questioned whether the virus could be resistant to complement-mediated lysis as in the case of certain other viruses (34,35, 36). To test the resistance of SARS-CoV-2 to virion lysis by NHS, we incubated SARS-CoV-2 virions with NHS and ribonuclease A (RNase A) and then determined the ability of RNAse A to access the genomic RNA and degrade virions upon virolysis (Fig. 3*A*). In control studies with SARS-CoV-2 treated with the detergent Triton-X instead of serum, qPCR detected negligible genomic viral RNA as expected (Fig. 3*B*). Conversely, the level of SARS-CoV-2 RNA was not statistically reduced when virus treated with NHS (1 to 10%) or with a human convalescent SARS-CoV-2 serum (10%) was cross-compared with untreated virus (Fig. 3*B*). We also tested NHS supplemented with rCL-11 in a final concentration (1 to 10 µg/mL; 9.52 to 95.24 nM) to optimize the level of bound CL-11 by SARS-CoV-2 as determined in earlier experiments (Fig. 1 *A* and *B*). However, NHS enriched with CL-11 had no detectable effect on virolysis compared with nonenriched serum (Fig. 3*C*).

In addition to testing virolysis, we ran infectivity assays to see whether NHS (sero-negative for SARS-CoV-2 spike antibody as determined by ELISA) impacted the ability of SARS-CoV-2 to infect cells and generate infectious virus. Here, we exposed Vero cells to a fixed amount of SARS-CoV-2 that had been pretreated with NHS or heat-inactivated NHS (HI NHS). No loss of SARS-CoV-2 infectivity after exposure of virus to as much as 25% NHS was evident when cross-compared with exposure to HI NHS (Fig. 3*D*). Resistance to serum-mediated virolysis (Fig. 3 *B* and *C*) was one possible explanation for how infectivity of SARS-CoV-2 was unimpeded in our experiment (Fig. 3*D*). We went on to explore another possibility: that CL-11 binding could have had a compounding effect on viral infectivity.

### Infectivity of SARS-CoV-2 Is Enhanced by Bound CL-11.

**Fig. 4.**
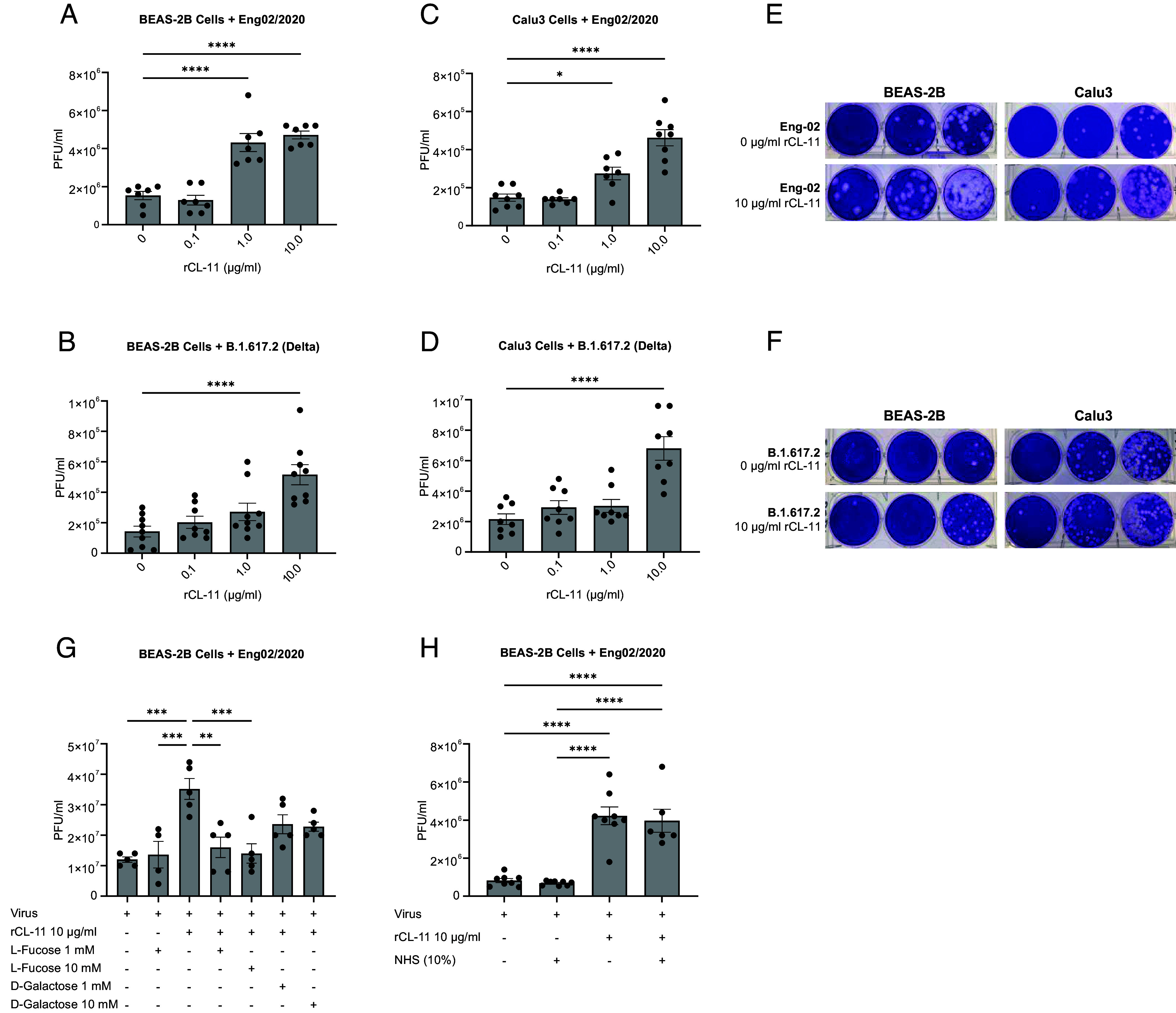
SARS-CoV-2 opsonized with CL-11 drives increased infectivity of respiratory epithelial cells. (*A* and *B*), Enhancement of BEAS-2B cell infection with SARS-CoV-2 variants pretreated with rCL-11 (0.1, 1.0, and 10.0 µg/mL) or media for 1 h at 37 °C. (*A*) England/02/2020 (MOI 0.005) and (*B*) B.1.617.2 (MOI 0.05). (*C* and *D*), Enhancement of Calu-3 cell infection with SARS-CoV-2 variants pretreated with rCL-11 (0.1, 1.0, and 10.0 µg/mL) or media for 1 h at 37 °C. (*C*) England/02/2020 (MOI 0.05) and (*D*) B.1.617.2 (MOI 0.05). Virus titers in the supernatant after 24 h determined by plaque assay. Data are pooled from two (*A*, *C*, *D*) or three (*B*) independent experiments and represent the mean ± SEM of triplicate or quadruplicate biological samples. Each group compared to 0 µg/mL rCL-11 by one-way ANOVA with Dunnett’s multiple comparison test, *P < 0.05, and ***P < 0.001. (*E* and *F*), Representative images of crystal violet stained plaque assay plates of BEAS-2B or Calu-3 cells infected with either England/02/2020 (*E*) or B.1.617.2 (*F*) or opsonized with 10 mg/mL rCL-11. (*G*) L-fucose inhibits the enhancement of SARS-CoV-2 (England/02/2020) infectivity driven by CL-11 in BEAS-2B cells. England/02/2020 (MOI 0.05) pretreated with media or with rCL-11 (10.0 µg/mL) in the presence or absence of L-fucose or D-galactose (1- or 10 mM final concentration) and virus titers determined as above. (*H*) Infectivity of SARS-CoV-2 (England/02/2020) opsonized with CL-11 is not inhibited by NHS. Quantification of SARS-CoV-2 England/02/2020 in the supernatant of BEAS-2B cells 24 h after infection with virus (MOI 0.005) pretreated with media or with rCL-11 (10.0 µg/mL) spiked with or without 10% NHS as detailed above. (*G* and *H*) Data are representative of two independent experiments with the mean ± SEM of five (*G*) and at least six (*H*) biological samples. **P < 0.01 and ***P < 0.001 determined by one-way ANOVA with Tukey’s multiple comparison test.

Certain human pathogens have adapted to complement allowing the microorganisms to escape destruction and promote tissue invasion (37,39, 40). We constructed an assay where RECs were incubated with SARS-CoV-2 that had been coated or not with rCL-11 over a range of predetermined concentrations up to 10 µg/mL, followed by quantification of virus released into the culture supernatant using a plaque forming assay in Vero cells. Remarkably, infectivity of the rCL-11-opsonized SARS-CoV-2 (England 02/2020) was up to 3.2-fold higher than for nonopsonized virus, based on the titer of virus released from infected bronchial epithelial BEAS-2B cells engineered to express ACE2 (Fig. 4 *A* and *E*). We also observed that lung epithelial Calu-3 cells, which naturally express ACE2 and entry cofactors TMPRSS2 and TEMPRSS4 (41), exhibited a similar degree of susceptibility to rCL-11-treated virus as BEAS-2B (Fig. 4 *C* and *E*). Likewise, we detected a threefold to fourfold enhancement of infection with the highly transmissible and infectious B.1.617.2 variant of SARS-CoV-2 (42, 43) in BEAS-2B and Calu-3 cells respectively (Fig. 4 *B*–*F*). Generally, the amplifying effect of rCL-11 was dose-dependent with a peak effect between 1 µg/mL and 10 µg/mL (Fig. 4 *A*–*F*). The reproducibility of the opsonizing effect of rCL-11 for different cells and different viral strains is indicative of conserved virus-expressed glycans critical for CL-11 binding (44).

To corroborate the importance of carbohydrate-recognition for CL-11 to enhance SARS-CoV-2 infectivity, we preincubated rCL-11 with monosaccharide to saturate the CRD on rCL-11 before mixing with the virus. We determined that preincubation of rCL-11 with L-fucose (1 mM or 10 mM) fully ablated the enhancing effect of CL-11 (Fig. 4*G*). D-galactose, which has lower binding affinity for CL-11 compared to L-fucose (11) had a partial blocking effect (Fig. 4*G*). These experiments indicated that interaction via the CRD of CL-11 rather than the CLD was primarily responsible for driving the enhanced infectivity of SARS-CoV-2.

The above infectivity experiments were conducted in the absence of exogenous complement; so, to understand whether viral infectivity was still enhanced by CL-11 when complement was present we performed assays in which rCL-11 had been spiked with NHS (10%) before mixing with SARS-CoV-2. This ensured adequate provision of complement factors (as indicated by Fig. 2) alongside rCL-11 and virus. We observed that rCL-11-enhanced viral infectivity was not dependent on or limited by the provision of complement excess in this setting (Fig. 4*H*).

### Respiratory Epithelial Cells Infected by SARS-CoV-2 Are Sensitive to Cell-Autonomous Complement.

**Fig. 5.**
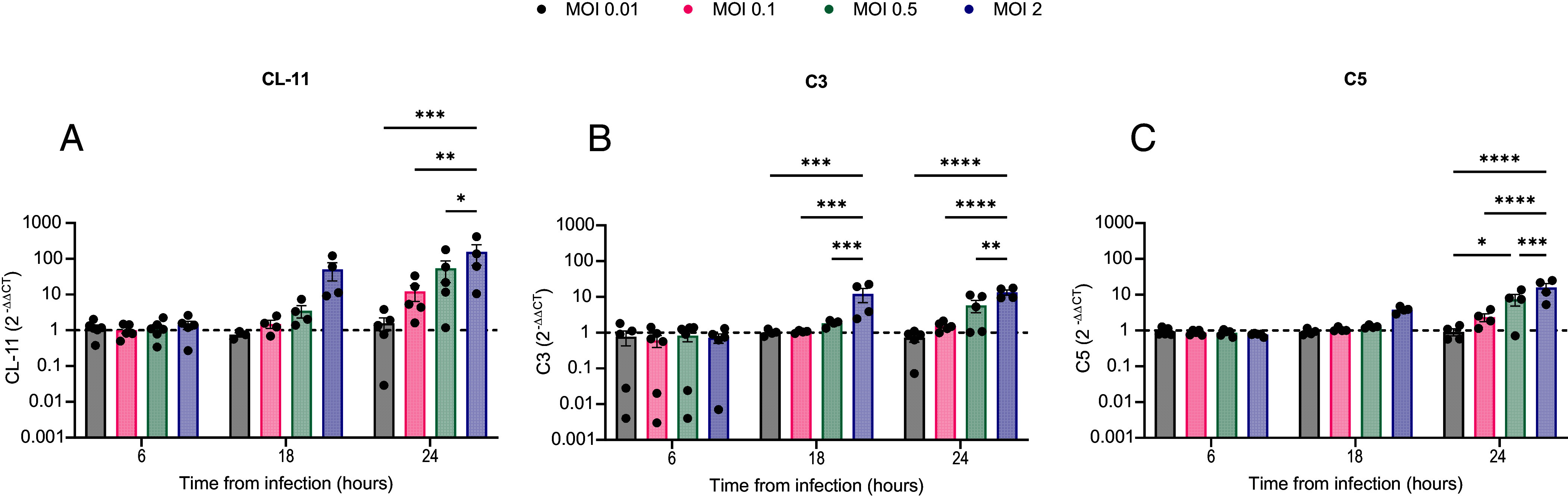
SARS-CoV-2 infection of bronchial epithelial BEAS-2B cells drives CL-11 and complement gene expression. (*A*–*C*) Fold mRNA induction (2^–∆∆Ct^ method) of (*A*) CL-11, (*B*) C3 and (*C*) C5 (by qRT-PCR) after infection of BEAS-2B cells with SARS-CoV-2 (England/02/2020) over a range of MOIs (0.01, 0.1, 0.5, and 2.0) at 6, 18, and 24 h pi. Data represent mean ± SEM from n = 4 independent experiments. Each group (within each time point) was compared by two-way ANOVA with Tukey’s multiple comparison test. *P < 0.05, **P < 0.01, ***P < 0.001, and ****P < 0.0001.

**Fig. 6.**
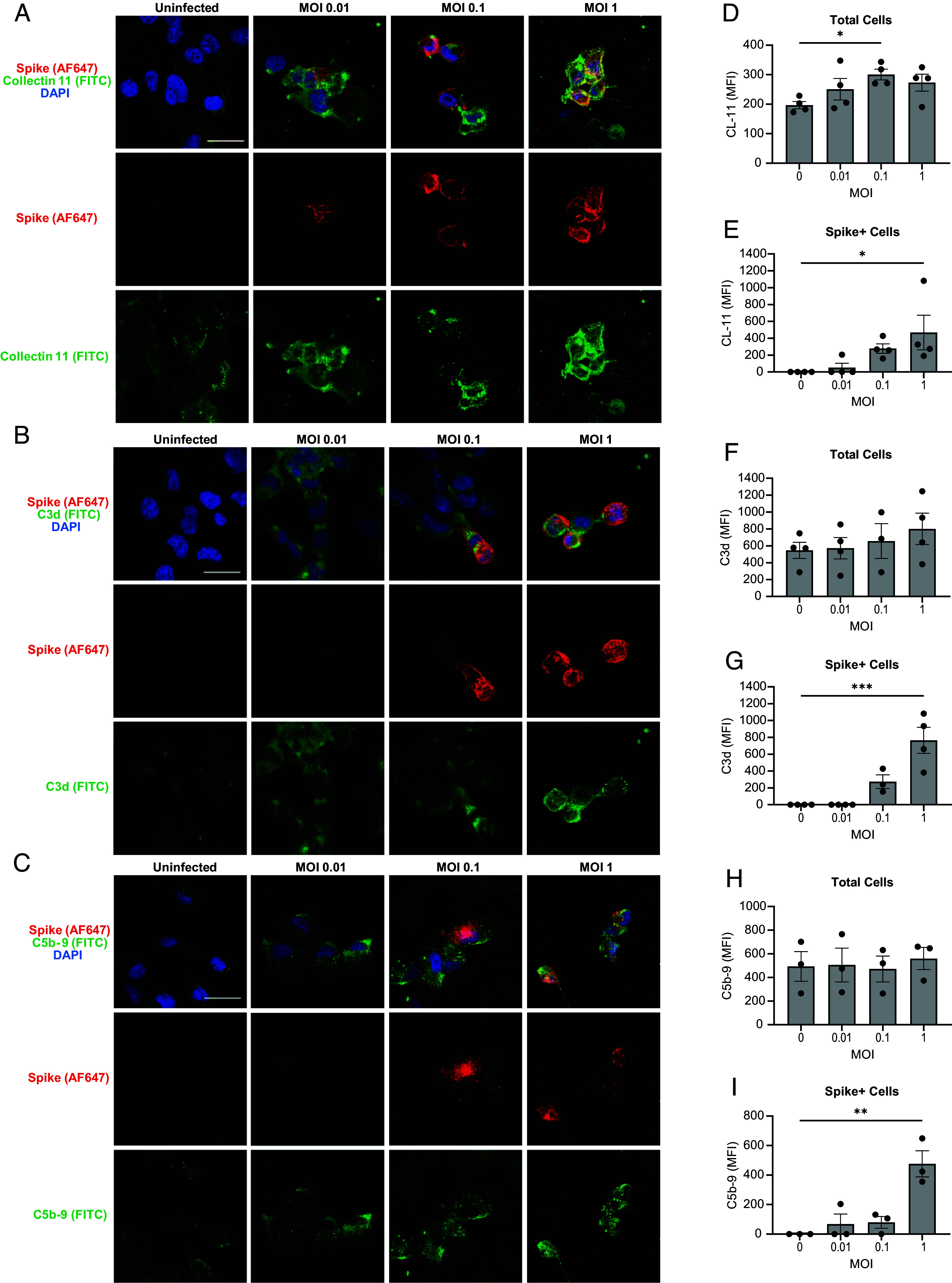
Bronchial epithelial BEAS-2B cells infected by SARS-CoV-2 are sensitive to cell-autonomous complement. (*A*, *B*, and *C*) Representative confocal microscopic images of BEAS-2B cells, mock, uninfected at 24 h after plating and 24 h after infection with SARS-CoV-2 (England/02/2020) over a range of MOIs (0.01, 0.1, and 1.0) and stained for cell surface spike protein (red), DAPI (blue) and either (*A*) cell surface CL-11 (green), (*B*) cell surface C3d (green) or (*C*) cell surface C5b-9 (green). Original magnification: × 60. (Scale bar: 30 µm.) Bar graphs show quantification of the MFI (mean ± SEM) from four independent experiments with a minimum of five images per condition per experiment for (*D*) CL-11 on all (total) cells imaged, (*E*) CL-11 on spike+ cells, (*F*) C3d deposited on all cells, (*G*) C3d deposited on spike+ cells, (*H*) C5b-9 on all cells and (*I*) C5b-9 on spike+ cells. Statistical comparisons are made by one-way ANOVA with Dunnett’s multiple comparisons between mock (uninfected) and SARS-CoV-2 infected conditions at each MOI (*D*, *E*, *F*, *G*, *H*, and *I*). *P < 0.05, **P < 0.01 and ***P < 0.001.

We envisaged that locally produced CL-11 interacting with SARS-CoV-2 facilitates viral infectivity at the portal of entry and, in turn, virus-infected cells drive complement activation through CL-11 binding. Supporting this hypothesis, we found that BEAS-2B cells infected with SARS-CoV-2 markedly upregulated gene expression of CL-11 and the core complement components C3 and C5. This upregulation was both time-dependent and proportionate to viral dose (MOI) (Fig. 5 *A*–*C*). Cell surface detection of CL-11 and C3d proteins by confocal microscopy (of nonpermeabilized cells) was most evident at higher virus MOIs (Fig. 6 *A* and *B* respectively), notably associated with the subpopulation of BEAS-2B cells expressing cell surface SARS-CoV-2 spike (spike^+^, Fig. 6 *E* and *G*, respectively). Colocalization of cell-surface staining for SARS-CoV-2 spike protein and CL-11 (Fig. 6 *A*, *Top* row) or C3d (Fig. 6 *B*, *Top* row) was partial, meaning that the area stained positive for CL-11 and complement extended beyond the foci where spike protein was detected, to involve a larger surface area of virus-stressed cells. Membrane attack complex (C5b-9) formation secondary to complement activation was detected at the higher MOI of SARS-CoV-2, in spike^+^ expressing cells compared to uninfected cells (Fig. 5*I*). Collectively, these observations suggest that cell stress in general and cell-surface presentation of SARS-CoV-2 spike protein in particular induced CL-11 and complement deposition following viral infection.

### CL-11 Enhances SARS-CoV-2 Infection and Initiates the Lectin-Complement Pathway in Primary Respiratory Epithelial Cells.

To strengthen the physiological relevance of our findings, we employed an in vitro model that closely recapitulates the human respiratory epithelium. Specifically, we established cultures of primary human bronchial epithelial cells (HBECs) isolated from three healthy donors that were fully differentiated at the air–liquid interface (ALI).

**Fig. 7.**
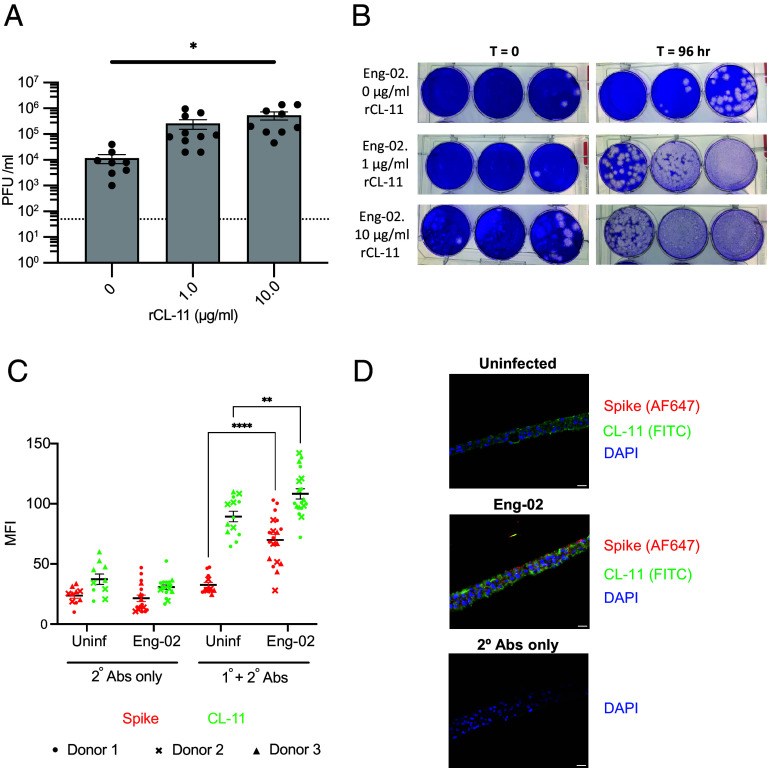
CL-11 modulates SARS-CoV-2 infection and initiates the lectin-complement pathway in primary HBECs. (*A*) Apical SARS-CoV-2 (England/02/2020) virus titers 96 h postinfection from HBEC ALI cultures following 1 h preincubation with recombinant CL-11 (0, 1.0, 10.0 µg/mL) at 37 °C. Data represent mean ± SEM from three donors (≥2 replicate cultures/donor). *P < 0.05 by one-way ANOVA with Dunnett’s correction to mock uninfected. (*B*) Representative plaque assay images from apical washes at 0 and 96 h postinfection. (*C*) Quantification of immunofluorescence staining for CL-11 (green), spike protein (red), and DAPI (blue) in mock (uninfected) and SARS-CoV-2-infected HBEC ALI cultures at 96 h. MFI from three donors (≥3 images/condition); analyzed by two-way ANOVA with Dunnett’s multiple comparisons to mock. **P < 0.01 and ***P < 0.001. (*D*) Representative cross-sectional images of HBEC ALI cultures (mock and infected, 96 h post infection), stained for CL-11 (green), spike (red), and DAPI (blue); secondary antibody control shown below. (Scale bar: 100 µm); original magnification ×200.

Strikingly, infection with SARS-CoV-2 (England 02/2020) opsonized with rCL-11 resulted in a 1.0 to 1.5 log_10_ increase in viral titers at 96 h postinfection, as measured by plaque assay of apical wash fluid, compared to infection with virus alone (Fig. 7 *A* and *B*). In parallel, we analyzed surface detection of CL-11 and spike protein by immunofluorescence in nonpermeabilized HBECs. We found that HBECs infected with SARS-CoV-2 demonstrated increased binding of CL-11 in cells expressing spike at 96-h post infection when compared with uninfected cells. (Fig. 7 *C* and *D* and *SI Appendix*, Fig. S3).

**Fig. 8.**
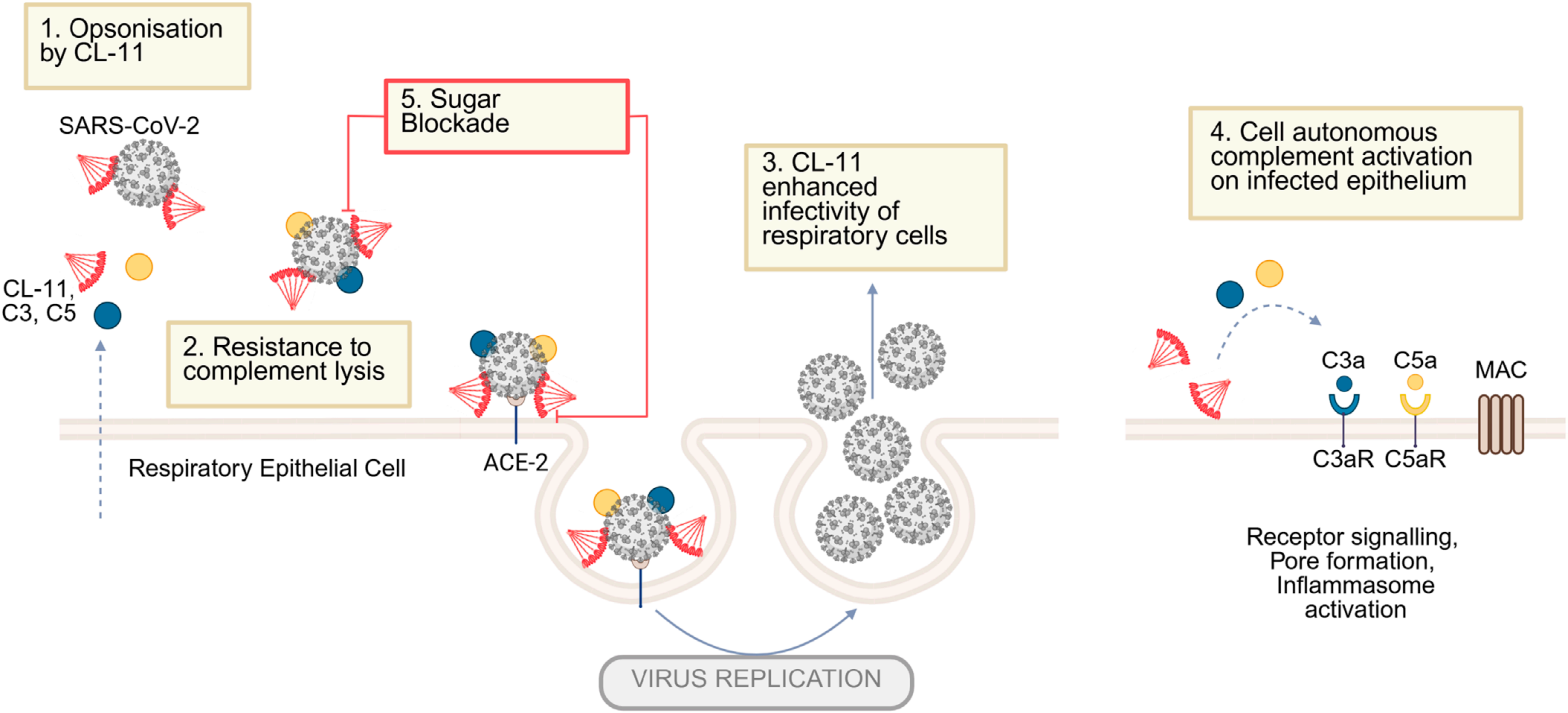
Schematic summarizes the key findings of the study demonstrating CL-11 modulation of REC invasion and injury by SARS-CoV-2. RECs are a source of CL-11, a secreted C-type protein interacting with glycan motifs on microbial and mammalian structures to stimulate complement activation. Our study demonstrates: (1) SARS-CoV-2 binds CL-11 and triggers complement activation to form membrane attack complex (MAC); (2) the virus however is resistant to lysis; (3) CL-11-opsonized virus exhibits enhanced infectivity, i.e., increased release of virus into the extracellular space of virus-infected HBECs by 1 to 1.5 log_10_ PFU/mL; (4) virus-infected RECs demonstrate cell-autonomous deposition of CL-11 and form complement products typically associated with inflammasome activation and membrane injury; (5) the cycle of enhanced infectivity can be interrupted by L-fucose given to saturate the carbohydrate-recognition domain of CL-11.

These data, collectively with those in Figs. 4 and 6 *A*, *B*, and *C*, indicate that CL-11 has the capacity to enhance SARS-CoV-2 infection of human respiratory epithelial cells and suggest that virus infection promotes CL-11 and complement deposition (Fig. 8).

## Discussion

CL-11 detects a wide range of pathogens and in this respect SARS-CoV-2 is no exception. In our study, binding to immobilized SARS-CoV-2 spike trimer protein occurred through the CRD of rCL-11, as typical for a C-type lectin and anticipated by our earlier structural modeling of N-glycosylation sites on the spike trimer (25). Predictably, SARS-CoV-2 triggered complement activation in the presence of normal serum that was sero-negative for SARS-CoV-2, coating the virus with C3b and MAC and liberating C3a and C5a. Yet, in virolysis assays where SARS-CoV-2 was exposed to serum (1 to 10%) no increase in lysis occurred compared to treatment with heat-inactivated serum, even with rCL-11 enriched serum. Furthermore, there was no evidence for loss in infectivity of SARS-CoV-2 when treated with serum (0 to 25%) in accord with resistance of the virus to lysis. Remarkably, pretreatment of virus by rCL-11 (1 to 10 µg/mL) increased virus infectivity by a mechanism independent of complement but abolished by blockade of carbohydrate-recognition by the lectin. Strikingly, respiratory epithelial cells infected by SARS-CoV-2 were vulnerable to self-injury by CL-11 and complement produced by those cells. Thus, an intrinsic pathogenic role of CL-11 exploited by SARS-CoV-2 is supported by these findings.

This combination of viral enhancement by CL-11 and resistance to lysis seemed to be conserved in successive variants of SARS-CoV-2, as shown here for the First Wave and Delta Wave variants. The perceived survival advantage may contribute to maintenance of SARS-CoV-2 load in the respiratory mucosal compartment, as has been observed equally in vaccinated and unvaccinated individuals (3, 4). While MBL, another major collectin protein bound by SARS-CoV-2 (26), also can enhance infectivity of some viruses, e.g., Ebola virus (5), the expression of MBL is exclusive to the liver and the circulation and hence it is unlikely to impact the infectivity of SARS-CoV-2 at the point of contact with respiratory epithelium. We would therefore argue that viral persistence, at least in the respiratory tract, is more likely to be modulated by CL-11 than MBL.

Pathogens adapted to evade lysis by MAC (8, 45) employ novel proteins or capture host proteins that inhibit C3 convertases or block subsequent MAC assembly. For example, the NS1 protein of Flaviviruses leads to the recruitment of host complement-regulatory proteins either inhibiting the classical or alternative pathways of complement activation or the formation of MAC (46). Nipah virus encodes a protease activity capable of cleaving C3b (34). HIV-1 can adsorb human GPI-anchored complement-regulatory proteins for protection against C3 cleavage and MAC formation (35). We propose it is selective pressure imposed by complement in a zoonotic reservoir that has caused SARS-CoV-2 to evolve a mechanism conferring relative resistance to elimination by complement. It is entirely feasible that exposure to the complement system, for example in bats (47, 48), may be a factor in the evolution of progenitor viruses before SARS-CoV-2 transmission to humans.

Early host defenses at epithelial surfaces are known to impact pathogen spread and disease sequelae (49). Our data for SARS-CoV-2 provide a previously unrecognized example revealing how locally produced CL-11 perversely enhances infectivity of RECs. This is distinct from other pathogens (e.g., HIV-1 and *Escherichia coli*) where it is opsonization by C3b that promotes intracellular invasion (8, 50). We found no evidence for such a mechanism here because SARS-CoV-2 incubated with rCL-11 in the presence of 10% NHS did not enhance viral infectivity over that with rCL-11 alone. Consequently, C3b opsonization of SARS-CoV-2—though it occurs—was nonessential for rCL-11-driven enhancement of infectivity in our system. Hence other properties of CL-11 require consideration. For instance, the multivalent structure of CL-11 oligomers and ability of the lectin to bind with both viral and mammalian glycan ligands may permit cross-linking between virus and epithelial cell surfaces. Possibly viral internalization or processing by cells expressing the ACE2 host-cell attachment receptor could be enhanced by this means. The property to usurp CL-11 may be especially important for Wuhan-like viruses with lower affinity for ACE2 (51, 52) to establish infection in respiratory epithelia that express lower levels of ACE2 compared to other tissues such as heart and kidney (53). We suggest that complement-resistance combined with enhanced infectivity mediated by CL-11 contributes to the pathogenicity of SARS-CoV-2 infection.

Our data reveal that cell-autonomous membrane injury by CL-11 and complement is a feature of SARS-CoV-2 infected RECs, involving foci where spike protein is expressed but also extending to areas where no spike protein is detected consistent with the generalized response of epithelial cells undergoing metabolic or physical stress (13, 14). This infers that both spike-associated and stress-associated glycans are targeted by CL-11. Thus, lectin complement pathway activation is an intrinsic component of the epithelial response to cell stress, here contributing to cell injury induced by SARS-CoV-2 infection. Alternative pathway recruitment amplifying the quantity of C3b deposited on RECs is not excluded by our data. Indeed, the secretome of bronchial and alveolar epithelial cells contains factors D and B (54), which are key components of the alternative pathway and are brought into play by the lectin-pathway enzyme MASP-3 acting on factor D (55, 56). In that scenario, both the lectin and alternative pathways would participate in epithelial damage initiated by viral invasion followed by cell-membrane pore (MAC) formation, C3aR and C5aR engagement, and downstream intracellular signaling.

The data presented here and in an independent report showing immobilized SARS-CoV-2 spike protein trimer can deposit CL-11 from normal serum (57), along with the early prediction of glycan interaction sites on the spike trimer (25), robustly support a role for CL-11 interacting with SARS-CoV-2. Our data significantly extend these findings with important insights into SARS-CoV-2 pathogenesis, using rCL-11 to probe the interaction with virus and immobilized spike protein and with cell-membrane-expressed spike protein. This evidence of SARS-CoV-2 pathogenicity and induced host cell injury offer a functional interpretation of the reported genetic link between CL-11 and adverse clinical outcome in COVID-19 (24).

Our results have implications for therapeutic strategy. Both mannosylated and fucosylated proteins are present on the SARS-CoV-2 spike protein (29) as well as on host-cell structures (58, 59), with high-mannose ligands being particularly prominent on the spike protein (29). The terminal mannose disaccharides of oligomannose glycans form high-avidity natural ligands for the CRD of the collectin (31). Additionally, the core-fucosylated ligands identified on the spike protein (29) may be recognized by CL-11. In support of this, CL-11 has been shown to bind certain N-glycans containing core fucosylation in glycan array studies (31), although it remains unclear whether such glycans are accessible on the fully glycosylated spike protein. Here, we show in complexes formed between the CRD of CL-11 and L-fucose that the carbohydrate-binding site is occupied by two such monosaccharides, the second L-fucose unusually occupying the framework structure of the CRD away from the Ca^2+^-binding site containing the first L-fucose. This unexpected feature of L-fucose interacting with both subsites of CL-11 justifies the use of L-fucose to block CL-11 binding both to natural oligomannose or complex fucosylated ligands that may be present on SARS-CoV-2 and injured RECs. Furthermore, supraphysiological doses of L-fucose have proven effective and safe in murine studies (60, 61) and human studies (62, 63), the former demonstrating effective CL-11 blockade in hypoxic tissue injury (60), thus supporting the feasibility for clinical translation.

In conclusion, our results advance the notion of CL-11 as a first-line sensor of innate immunity at the portal of entry, aberrantly maintaining the cycle of respiratory epithelial infection by SARS-CoV-2 and directing collateral injury (by intrinsic complement) against infected cells. The potential for simple interventions to prevent CL-11 interacting with viral and cell-expressed glycan ligands merits further consideration.

## Materials and Methods

Full details on methods are provided in *SI Appendix*, *SI Materials and Methods*.

### Recombinant Proteins, Serum, and Antibodies.

rCL-11 was expressed in mammalian CHO cells and purified as previously described (31). A wheat-germ expressed rCL-11 (for comparative evaluation by ELISA) was purchased from Abnova. Unless specifically stated, CHO expressed rCL-11 was used in all experiments. All SARS-CoV-2 spike proteins were purchased from AcroBiosystems. NHS pooled from male AB blood donors (pre-COVID) and confirmed SARS-CoV-2 spike seronegative by ELISA was obtained from NHS Blood and Transplant Services. A convalescent serum sample from a SARS-CoV-2 infected human donor, exhibiting a high anti-spike antibody titer by ELISA, was included as a control.

For some experiments NHS was heat inactivated (HI) (30 min at 56 °C) prior to use. Full details of antibodies used are listed in *SI Appendix*, Table S2.

### Cell Lines and SARS-CoV-2 Viruses.

HEK293T, Vero E6-TMPRSS2, and Calu-3 were grown in Dulbecco’s modified Eagle’s medium (DMEM, Gibco) supplemented with GlutaMAX, 10% fetal bovine serum (FBS) and 100 U/mL penicillin and 100 μg/mL streptomycin (Pen/Strep; Gibco). BEAS-2B-ACE2 (gift of Stuart Neil, King’s College London, UK) were grown in RPMI 1614 supplemented with GlutaMAX (Gibco), 10% FBS, and Pen/Strep (Gibco) and maintained with 5 μg/mL blasticidin (InvivoGen) selection.

SARS-CoV-2 strain England 02/2020/407073 (referred to as England 02/2020) was obtained from Public Health England and strain B.1.617.2 (Delta variant) was a gift of Wendy Barclay, Imperial College London, UK. Virus propagation and titration by plaque assay is described in *SI Appendix*, *SI Materials and Methods*. All work with infectious SARS-CoV-2 was carried out in a Containment Level 3 facility (Health and Safety Executive approvals, CBA1.295.20.1 and GM386/20.2).

### Inactivation of SARS-CoV-2 by UV Irradiation.

SARS-CoV-2 (England 02/2020) was exposed to UV-C (254 nm emission) for 5 min at a distance of 7.62 cm. All UV inactivated virus stocks were titrated by plaque assay to ensure no replicative virus remained.

### Binding of rCL-11 to Immobilized SARS-CoV-2 Proteins.

Nunc MaxiSorp 96-well plates were coated with either UV-inactivated SARS-CoV-2 England/02/2020 or with SARS-CoV-2 spike protein Wuhan or Omicron BA.1 (AcroBiosystems) in carbonate/bicarbonate buffer for 18 h at 4 °C. Plates were then washed, blocked, and incubated with rCL-11 (CHO cell produced) as described in *SI Appendix*, *SI Materials and Methods*. Bound rCl-11 was detected with rabbit anti-human CL-11 antibody and revealed by incubation with horseradish peroxidase conjugated goat anti-rabbit IgG and 3,3’,5,5’-tetramethylbenzidine (TMB) substrate. Absorbance was read at 450 nm. BSA was used as a control protein. The molecular mass of rCL-11 was 105 kDa corresponding to a trimer of polypeptides (based on SDS gels), the most abundant form of CL-11.

For the sugar blockade experiments, 96-well Nunc MaxiSorp plates were coated with SARS-CoV-2 Wuhan spike protein (AcroBiosystems) at 2 µg/mL in carbonate/bicarbonate buffer, washed and blocked (as above). Plates were then incubated with rCL-11 (3 μg/mL final concentration) and L-fucose or D-galactose (0 to 200 mM) or with 20 mM EDTA for 18 h at 4 °C and specific CL-11 binding detected as above.

To compare the binding of rCL-11 produced in-house from transfected CHO cells (31) with wheat-germ expressed rCL-11 (Abnova), the ELISA protocol described by ref. 26 was used.

### Binding of rCL11 to Cell Surface Expressed SARS-CoV-2 Spike Protein.

HEK293T cells were transfected with SARS-CoV-2 Wuhan spike in pcDNA3.1 or the empty vector as previously described (64). At 48 h post transfection, cells were harvested, washed in PBS containing 2% FBS and 1 mM EDTA (FACS buffer) and incubated with rCL-11 at 25 µg/mL in PBS containing 2 mM CaCl_2_ or with PBS alone for 1 h at RT. In some experiments, rCL-11 was preincubated with either PBS, L-fucose (10 mM), or EDTA (10 mM) prior to incubation with spike or empty vector transfected HEK293T cells for 30 min at RT. Full staining and data acquisition details used to quantitate cell surface SARS CoV-2 spike protein and bound rCL-11 by flow cytometry is given in *SI Appendix*.

### Production of the CRD of CL-11.

This is described in detail in *SI Appendix*, *SI Materials and Methods*.

### Crystallization and Structure Determination.

Crystals were grown using the sitting-drop vapor diffusion method and diffraction data collected as detailed in *SI Appendix*.

### Complement Activation Assay.

To measure activation of C3 and C5, live SARS-CoV-2 was incubated with NHS and levels of the cleavage products C3a-desArg and C5a determined by ELISA (Hycult Biotech kit) according to the manufacturer’s instructions.

### Complement Deposition Assay.

Activation and deposition of NHS derived C3d and C5b-9 by immobilized UV-inactivated virus (England/02/2020) was determined by ELISA as detailed in *SI Appendix*.

### Viral Lysis Assay.

Complement-sufficient NHS (0 to 10% final concentration) or an IHS (10% final concentration) was mixed 10^3^ PFU of SARS-CoV-2 and RNAse A (1 mg/mL,Thermofisher) for 2 h at 37 °C. The reaction was stopped on ice and samples frozen 18 h at −20 °C to rupture damaged virions. Samples were then thawed, mixed with fresh RNAse A (1 mg/mL, Thermo Fisher Scientific) and incubated for 1 h at 37 °C, followed by incubation with Proteinase K (1 mg/mL, NEB) for 20 min at 37 °C to remove RNAse A. Residual viral RNA was extracted and purified using the QiAMP Viral RNA extraction kit (QIAGEN) and quantified by RT-qPCR using a SARS-CoV-2 (2019-nCoV) CDC qPCR E primer and probe assay (Integrated DNA Technologies, #10006804). PCRs were performed using a QuantStudio-5 Real-Time PCR machine (Applied Biosystems) and analyzed using QuantStudio Design and Analysis Software v1.5.2 (Applied Biosystems). Viral E genome equivalent copies were determined by standard curve, using an E gene standard.

### Serum Blocking Infectivity Assay.

10^4^ PFU of SARS-CoV-2 (England/02/2020) was incubated for 1 h at 37 °C with an equal volume of pooled NHS (confirmed SARS-CoV-2 spike antibody negative by ELISA) at 0 to 25% (final NHS concentration) or with 25% HI NHS (56 °C for 30 min). Serial dilutions of NHS-virus mixtures were then titrated for infectious virus by plaque assay as stated above.

### Virus Infections.

BEAS-2B-ACE2 and Calu-3 cells seeded in appropriate media were incubated for 1 h at 37 °C with mixtures of virus (equivalent to an MOI of 0.005 or 0.05 dependent upon the virus and cell line indicated in the figs) that had been preincubated for 1 h at 37 °C with rCl-11 at either 0, 0.1, 1.0, or 10.0 mg/mL (final concentration). The virus/rCl-11 mixture was then aspirated, the cells washed and reincubated for 24 h at 37 °C in fresh 2% HI FBS media. Supernatants were then collected to determine infectious virus titer by plaque assay.

### RT-qPCR.

RNA from infected and uninfected cells was extracted using a Qiagen RNeasy minikit (Qiagen; 74106) following the manufacturer’s instructions. Ten microliter of each extracted RNA was used to synthesize cDNA using a High-Capacity cDNA Reverse Transcription kit (Applied Biosystems, 4368813). RT-qPCR was performed using TaqMan Universal Master Mix (Applied Biosystems, 4304437) with specific TaqMan probes to quantify *COLEC11* (Hs00388161_m1), *C3* (Hs00163811_m1), *C5* (Hs01004342_m1), and *GAPDH* (Hs99999905_m1) all purchased from Applied Biosystems. RT-qPCRs were performed according to the manufacturers instructions using a QuantStudio-5 Real-Time PCR machine (Applied Biosystems) and QuantStudio Design and Analysis Software v1.5.2 (Applied Biosystems). Relative quantification was performed using the 2^−ΔΔCT^ method (65) with sample data first normalized to GAPDH mRNA levels and then to values from uninfected cell control samples.

### Confocal Microscopy.

BEAS-2B-ACE2 cells (2.5 × 10^4^ cells per coverslip) were infected with SARS-CoV-2 England/02/2020 (MOI of 0, 0.01, 0.1, and 1) for 1 h at 37 °C, then washed and incubated for 24 h at 37 °C followed by fixation with 4% PFA. Fixed cells were blocked 30 min with PBS containing 20% normal sera (according to the species of the secondary antibody) then incubated with primary antibodies followed by an anti-species secondary antibodies (indicated in the figures) and thereafter with DAPI (Invitrogen) and mounted in PermaFluor (Epredia). Full staining details and a list of antibodies used are given in *SI Appendix*, *SI Materials and Methods*. Cells were imaged on a Nikon A1 inverted confocal (Eclipse Ti-E) microscope, using a Nikon Plan Apo lambda 60×/1.40 oil immersion lens. An average of 5 images were collected blind for each sample. All images were processed using Image J software (NIH) and quantitated as mean fluorescence intensity by circling cell surface spike^+^ or spike^−^ cells as described (66).

### Experimental Primary Cell Culture Model and Donor Details.

Donor 1: female, 62 y, Caucasian

Donor 2: male, 71 y, Caucasian

Donor 3: male, 62 y, Caucasian

Cryopreserved HBECs from healthy donors obtained from Epithelix and PromoCell were expanded and then seeded (2.12 × 10^5^ cells/cm^2^) onto collagen coated (30 µg/mL) tranwells and cultured submerged in Bronchial Epithelial Cell Growth Medium (Promocell) until confluent. Cells were then air-lifted and maintained with biweekly refeeding in differentiation medium (PromoCell) for 3 to 4 wk until beating ciliated cells and mucus were observed.

### Virus Infection of HBEC Cultures.

Prior to infection, the apical surface of the HBEC ALI culture was rinsed 3× with Hanks media, aspirated, and incubated for 1.5 h at 37 °C and 5% CO_2_ with mixtures of SARS-CoV-2 England 02/2020 (equivalent to an MOI of 0.1) that had been preincubated for 1 h at 37 °C with rCl-11 at either 0, 1.0, or 10.0 μg/mL (final concentration, as described above). The virus/rCl-11 mixture was then aspirated, the cells washed 3× with 200 μL Hanks media and reincubated for 96 h at 37 °C. The last wash was collected for the 0 time point for virus assay. After 96 h, 200 μL Hanks medium was applied to the apical surface, incubated 15 min at 37 °C, collected, and titered by plaque assay.

### Whole-Mount Immunostaining and Imaging.

SARS-CoV-2 infected and mock infected HBEC ALI cultures were washed 2× in PBS, fixed in 4% (w/v) paraformaldehyde in PBS (20 min at room temperature), rewashed, and processed for paraffin embedding. For immunofluorescence staining, 4 µm sections were deparaffinized followed by heat-induced antigen retrieval (1 mM EDTA, pH 8.0) prior to blocking in 2% BSA in PBS. Sections were stained with primary antibodies specific for SARS-CoV-2 spike protein and CL-11 overnight at 4 °C followed by species-specific secondary antibodies as listed in *SI Appendix*, Table S2. Nuclei were visualized with DAPI (Invitrogen). Images were processed using Image J software (NIH) and quantitated as mean fluorescence intensity.

### Ethical Permissions.

Approval for blood collection from a SARS-CoV-2 convalescent donor was obtained from the London Bridge Research Ethics Committee (REC number14/LO/1699).

### Statistical Analysis.

Statistical analysis was performed using PRISM Software version 10.1 (GraphPad). Comparisons between multiple groups were performed using either a one-way or two-way ANOVA with multiple comparison tests as described in the figure legends. Data show the mean ± SEM, with significance shown in the figures and levels defined as *P < 0.05, **P < 0.01, ***P < 0.001, ****P < 0.00001.

## Supplementary Material

Appendix 01 (PDF)

## Data Availability

All data generated or analyzed during this study are included in the article and/or *SI Appendix*. The atomic coordinates for CL-11 have been deposited in the Protein Data Bank (30) under PDB:95RK.
