# Supplementary material for: Glycan recognition by collectin-11 drives SARS-CoV-2 infectivity and membrane injury of respiratory epithelial cells
Source: Proc Natl Acad Sci U S A. 2025 Oct 24;122(43):e2521209122. doi: 10.1073/pnas.2521209122 (PMC12582335; doi:10.1073/pnas.2521209122)
Supplement: Supplementary file 1 — Appendix 01 (PDF) [file pnas.2521209122.sapp.pdf]

PNAS

Supporting Information for

Glycan recognition by collectin-11 drives SARS-CoV-2 infectivity and membrane injury of respiratory epithelial cells.

Anastasia Polycarpou[a1], Tara Wagner-Gamble[b,1], Roseanna Greenlaw[a,2], Lauren O'Neill[b,2], Varsha Kanabar[b,], Alanoud Alrehaili[c], Yusun Jeon[a], Jonathan Baker[d], Mona Bafadhel[d], Hataf Khan[b], Michael H. Malim[b], Marco Romano[a], Conrad A. Farrar[a], Dorota Smolarek[a], Rocio Martinez-Nunez[b], Katie J. Doores[b], Russell Wallis[c], Linda S. Klavinskis[b,3*] and Steven H. Sacks[a,3*]

[a] Peter Gorer Department of Immunobiology, School of Immunology and Microbial Sciences, King's College London, London SE1 9RT, UK.

[b] Department of Infectious Diseases, School of Immunology and Microbial Sciences, King's College London, London SE1 9RT, UK.

[c] Department of Respiratory Sciences, University of Leicester, Leicester, LE1 9HN, UK.

[d] King's Centre for Lung Health, School of Immunology and Microbial Sciences, King's College London, London SE1 9RT, UK

[1]These authors contributed equally to this work (in alphabetical order)

[2]These authors made substantial contributions to this work (in alphabetical order)

[3]These authors contributed equally to this work as senior authors (in alphabetical order)

[*]Corresponding authors, Linda Klavinskis and Steven Sacks

Email linda.klavinskis@kcl.ac.uk, steven.sacks@kcl.ac.uk

This PDF file includes:

Suporting Materials and Methods
Figures S1-S4
Tables S1-S2
SI References

Supporting Information text

Materials and Methods

Cell lines and SARS-CoV-2 viruses. HEK293T (ATCC CRL-3216), Vero E6-TMPRSS2 and BEAS-2B-ACE2 (kind gift of Stuart Neil, King's College London) and Calu-3 (ATCC HTB-55, kind gift of Jane McKeating, University of Oxford) were grown in Dulbecco's modified Eagle's medium (DMEM, GIBCO) supplemented with GlutaMAX, 10% fetal bovine serum (FBS) and 100 U/ml penicillin and 100 μg/ml streptomycin (Pen/Strep; Gibco). BEAS-2B-ACE2 (kind gift of Stuart Neil) were grown in RPMI 1614 (Gibco) supplemented with GlutaMAX, 10% FBS and Pen/Strep (Gibco) and maintained with 5 μg/ml blasticidin (InvivoGen) selection.

SARS-CoV-2 strain England 02/2020/407073 (referred to as England 02/2020) was obtained from Public Health England and strain B.1.617.2 (Delta variant) was a kind gift of Wendy Barclay, Imperial College London. Both isolates were propagated by infecting 60–70% confluent Vero E6-TMPRSS2 cells in T75 flasks. Supernatant was harvested 72 h post-infection after visible cytopathic effect, and filtered through a 0.22-μm filter (Sartorius) to eliminate debris, aliquoted and stored at −80℃. Infectious virus titer was determined by plaque assay using Vero E6-TMPRSS2 cells. All work with infectious SARS-CoV-2 was carried out in a Containment Level 3 facility (Health and Safety Executive approvals, CBA1.295.20.1 and GM386/20.2).

Inactivation of SARS-CoV-2 by UV irradiation. UV inactivation of virus was performed by exposure of SARS-CoV-2 (England 02/2020) at a maximal depth of 1.5mm in 6-well plates (Sarstedt) placed on ice, to a UV device (Jena Analytik) at 254 nm emission at a distance of

7.62cm for 5 min. All UV inactivated virus stocks were titrated by plaque assay to ensure no replicative virus remained.

Plaque assay. This was performed by infecting Vero-E6-TMPRSS2 cells with serial dilutions of SARS-CoV-2 for 1 h at 37°C. Subsequently, 2x overlay medium (DMEM with 2% FBS and 0.1% agarose) was added, and infected cells were fixed with 4% paraformaldehyde (PFA) at 72 h after infection and stained with crystal violet. Plaques were counted and virus titre determined.

Binding of rCL-11 to immobilized SARS-CoV-2 proteins. Nunc MaxiSorp 96-well plates were coated with either UV-inactivated SARS-CoV-2 England/ 02/2020 or with SARS-CoV-2 spike protein, Wuhan or Omicron BA.1 (AcroBiosystems) over a concentration range in carbonate/bicarbonate buffer for 18h at 4°C. The plates were washed four times with Tris buffered saline (10mM Tris, 145mM NaCl, 0.05% Tween-20, 2mM $CaCl_2$ pH7.4) before blocking with 0.1% bovine serum albumin (BSA) in Tris buffer for 1h at RT. Plates were then rewashed and incubated with rCL-11 (CHO cell produced) diluted in 10mM Tris, 145mM NaCl, 2mM $CaCl_2$ for 1h at RT. Plates were then washed and incubated with rabbit anti-human CL-11 antibody (1:1000, Abcam) for 1h at RT. Plates were re-washed and incubated with goat anti-rabbit IgG-horseradish peroxidase (HRP) antibody (1:3000, Cell Signalling Technologies) for 1h at RT. After washing, plates were developed with 3,3',5,5'-tetramethylbenzidine (TMB, Thermo Fisher Scientific) and absorbance read using a Hidex microplate reader (LabLogic) at 450nm. Values from blank wells were subtracted from sample wells. In certain experiments BSA (at the same molar concentration as rCL-11) was used as a control protein to rCL-11. The molecular mass of rCL-11 was 105 kDa corresponding to a trimer of polypeptides (based on SDS gels), the most abundant form of CL-11.

For the sugar blockade experiments, 96-well Nunc MaxiSorp plates were coated with SARS-CoV-2 Wuhan spike protein (AcroBiosystems) at 2μg/ml in carbonate/bicarbonate buffer for 18h at 4°C. Plates were then washed, blocked with 0.1% BSA (in Tris buffer) for 2h at RT and rewashed (as above). Plates were then incubated with rCL-11 (3μg/ml final concentration) and with either L-fucose, D-galactose or D-mannose over a titration range (0-100mM) or with 20mM EDTA, (all in 10mM Tris, 145mM NaCl, 2mM $CaCl_2$) for 18h at 4 °C. Plates were then washed, incubated with rabbit anti-human CL-11 IgG (1:1000, Abcam) for 1h at RT, re-washed and incubated with goat anti-rabbit IgG-HRP antibody (1:3000, Cell Signalling Technologies) for 1h at RT and developed with TMB (as described above).

To compare the binding of rCL-11 produced in-house from transfected CHO cells (1) with wheat-germ expressed rCL-11 (Abnova), the ELISA protocol described elsewhere (2) was used. In brief, nickel high binding plates (Thermo Scientific, 15442) were coated with recombinant SARS-CoV2 spike protein (AcroBiosystems) at 2μg/ml in Tris buffered saline (10mM Tris base, 150mM $NaCl_2$, 2mM $CaCl_2$, pH 7.5) for 1h at 20°C. The plates were washed three times with Tris-Tween buffer (Tris buffered saline containing 0.1% Tween) and blocked with 2% BSA in Tris-Tween buffer for 2h at 37°C. Plates were then washed three times (as above), followed by addition of rCL-11 (CHO cell or wheat-germ expressed) at 0-9μg/ml in Tris buffered saline for 1hr at 37°C. After three washes with Tris-Tween buffer, a rabbit anti-human CL-11 IgG (1:10000, Abcam) was added to wells and incubated for 1h at 37°C. Plates were subsequently washed three times prior to addition of a goat anti-rabbit IgG-HRP antibody (1:3000, Cell Signalling Technologies), for 1h at RT. Plates were developed by addition of TMB, then quenched with 1M H_2SO_4 and the absorbance was read at 450nm as indicated above. Values from blank wells were subtracted from sample wells.

Binding of rCL11 to cell surface expressed SARS-CoV-2 spike protein. HEK293T cells were transfected as previously described (3) with either pcDNA3.1 (empty vector) or with SARS-CoV-

2 Wuhan spike in pcDNA3.1 (kindly provided by Nigel Temperton, University of Kent). At 48 h post transfection, cells were harvested, washed in PBS containing 2% FBS and 1mM EDTA (FACS buffer) and 300,000 cells/sample were incubated with rCL-11 at 25μg/ml in PBS buffer containing 2mM $CaCl_2$ or with the cognate buffer alone (as control), shaking for 1h at RT. In some experiments, rCL-11 was preincubated with either PBS, L-fucose (10 mM) or EDTA (10 mM) prior to incubation with spike or empty vector transfected HEK293T cells for 30 min at RT. The cells were then washed, stained with live/dead brilliant violet (BV510, Invitrogen) for 30 min at 4°C, washed, and stained with rabbit anti-human CL-11 IgG (20μg/ml, Abbexa) directly conjugated to Alexa fluor647(AF647, Invitrogen Alexa Fluor™ antibody labelling kit) for 45 min at 4°C. Cells were washed, stained with human SARS-CoV-2 anti-spike mAb (25μg/ml, P008_108)(4) for 1h at 4°C followed by PE-rat anti-human IgG Fc (1:200, Biolegend) for 45 min at 4°C. Stained cells were washed, fixed with 4% paraformaldehyde (PFA) for 45 min at 4°C, rewashed, and acquired on a LSR Fortessa flow cytometer (BD). Data was analyzed using FlowJo software (version 10.1;TreeStar).

For the sugar blockade experiments rCL-11 was pre-treated with L-fucose or D-galactose (10mM) or EDTA (10mM) for 30 min at RT and then incubated with transfected HEK293T cells in PBS containing 2mM $CaCl_2$ for 1h at RT shaking. Cells were stained and analysed by flow cytometry as indicated above.

Production of the CRD of CL-11. The fragment of the cDNA encoding the CRD of CL-11 was amplified by PCR and cloned into the polylinker of expression vector pET28a. Cells were grown in Power Prime broth (Molecular Dimensions Limited, Newmarket, UK), induced during mid-log phase with IPTG (1 mM), and harvested after growth at 37°C for an additional 16 hours. Inclusion bodies were isolated and resuspended in 50 mM Tris–HCl pH 8.0, containing 6M guanidine-HCl and 5 mM dithiothreitol and protein was refolded by drop dilution into 50 mM Tris–HCl pH 8.0,

containing 9.6 mM NaCl, 0.4 mM KCl, 2 mM $MgCl_2$, 2 mM $CaCl_2$, 0.5 M arginine, 0.05% polyethylene glycol 3,550, 1 mM GSH and 0.1 mM GSSH at a final protein concentration of 0.1 mg/ml. Proteins were purified by ion exchange chromatography on a 2 mL Q-Sepharose column, using a 0.05 to 1 M gradient of NaCl in 20 mM Tris–HCl at pH 8.0, followed by gel filtration on a Superdex 75 16/60 column (GE Healthcare) in 20 mM Tris at pH 7.5 containing 50 mM NaCl and 2 mM CaCl2.

Crystallization and structure determination. Crystals were grown using the sitting-drop vapour diffusion method by mixing equal volumes (1.2 + 1.2 μL) of protein (10 mg/ml) and reservoir solution comprising 0.1 M Tris-acetate pH 8.5 containing 25% PEG High Smear (Molecular Dimensions, Newmarket, UK). Crystals were transferred to reservoir solution containing 15% (vol/vol) L-fucose (0.9 M), before storage in liquid nitrogen and were maintained at 100 K during data collection. Diffraction data were collected at beamline I04 at Diamond Light Source and were processed with xia2. Phases were determined by molecular replacement with Phaser (5) using the structure PDB: 4YLI as a search model. Models were optimized by using cycles of manual refinement with Coot (6) and refinement in Refmac5 (7), part of the CCP4 software suite (8), and in Phenix (9).

Complement activation assay. To measure C3a and C5a activation by SARS-CoV-2, 20μl NHS was incubated for 1 hr at 37°C with an equal volume of live SARS-CoV-2 (England/02/2020) from a 1:5 or 1:2 dilution series (as indicated) in PBS containing 2mM $CaCl_2$. The reaction was stopped, and virus inactivated by incubation with PBS containing 1%Tween-20 for 30 mins on ice. C3a-desArg and C5a levels were determined by ELISA (Hycult Biotech kit) according to the manufacturer's instructions.

Complement deposition assay. To measure activation and deposition of complement by SARS-CoV-2, Nunc MaxiSorp plates were coated with UV-inactivated virus (England/02/2020) in carbonate/bicarbonate buffer for 18h at 4°C. Plates were washed in TBS/Tween (10mM Tris, 145mM NaCl, 0.05% Tween-20, 2mM $CaCl_2$, pH 7.4) and blocked with 3% BSA in TBS/Tween for 2h at RT. NHS diluted (0–10%) in TBS plus 2mM $CaCl_2$ was added to plates and incubated for 1h at 37°C. The plates were washed again and bound C3d or C5b-9 detected using a monoclonal anti-C3d antibody (1:500, Abcam) or a monoclonal anti-C5b-9 antibody against the neoepitope (1:100, Agilent Dako) and incubated for 1h at RT. Plates were subsequently washed, developed with TMB and reaction quenched with 1M H_2SO_4. The absorbance was read using a Hidex microplate reader (LabLogic) at 450nm. Values from blank wells were subtracted from sample wells.

Viral lysis assay. Complement sufficient NHS (0-10% final concentration) or an IHS (10% final concentration) was mixed 10^3 PFU of SARS-CoV-2 and RNAse A (1mg/ml,Thermofisher) for 2 hr at 37°C. The reaction was stopped on ice and samples frozen 18 hr at -20°C to rupture damaged virions. Samples were then thawed, mixed with fresh RNAse A (1mg/ml, Thermo Fisher Scientific) and incubated for 1 hr at 37°C, followed by incubation with Proteinase K (1mg/ml, NEB) for 20 min at 37°C to remove RNAse A. Residual viral RNA was extracted and purified using the QiAMP Viral RNA extraction kit (QIAGEN) and quantified by RT-qPCR using a SARS-CoV-2 (2019-nCoV) CDC qPCR E primer and probe assay (Integrated DNA Technologies, #10006804). PCR reactions were performed using a QuantStudio-5 Real-Time PCR machine (Applied Biosystems) and analysed using QuantStudio Design and Analysis Software v1.5.2 (Applied Biosystems). Viral E genome equivalent copies were determined by standard curve, using an E gene standard kindly provided by Wendy Barclay, Imperial College London.

Serum blocking infectivity assay. To test for serum-induced virolysis by an infection based assay, 10^4 PFU of SARS-CoV-2 (England/02/2020) was incubated for 1 hr at 37 °C with an equal volume of pooled NHS (confirmed SARS-CoV-2 spike antibody negative by ELISA) at 0-25% (final NHS concentration) or with 25% HI NHS (56 °C for 30 min). Serial dilutions of NHS-virus mixtures were then titrated for infectious virus by plaque assay as stated above.

Virus infections. BEAS-2B-ACE2 were seeded in 12-well plates at $2x10^5$ cells/ well in complete RPMI media for 24 hr and Calu-3 cells were seeded in 24-well plates at 2.5 $x10^5$ /well for 48 hr in complete DMEM. The media was then removed, cells washed with 2% HI FBS containing media and incubated for 1 hr at 37°C with mixtures of virus (equivalent to an MOI of 0.005 or 0.05 dependant upon the virus and cell line as indicated in the data figs) that had been preincubated for 1 hr at 37°C with rCl-11 at either 0, 0.1, 1.0 or 10.0 mg/ml (final concentration). The virus/rCl-11 mixture was then aspirated and cells washed in 2% HI FBS containing media. Cultures were incubated for 24 hr at 37°C in fresh 2% HI FBS media. Supernatants were then collected and stored at -80°C prior to infectious virus titre determined by plaque assay.

Confocal microscopy. Coverslips in 24-well plates were pre-coated with poly-l-lysine for 18h at 4°C, then washed with PBS and seeded at a density of $2.5x10^4$ BEAS-2B cells per coverslip in 500ul complete RPMI media for 24 hr. Prior to infection, cells were washed with RPMI containing 2% HI FBS and then incubated with SARS-CoV-2 England/02/2020 (MOI of 0, 0.01, 0.1 and 1) for 1h at 37°C. Cells were then washed, replenished with RPMI containing 2% HI FBS and incubated for 24h at 37°C. Thereafter, cells were washed with PBS, fixed with 4% PFA for 30 min, washed again with PBS and then stored at 4°C until staining was performed. Fixed cells were blocked 30 min with PBS containing 20% normal sera (according to the species of the secondary antibody). For spike staining, PBS containing 1% BSA was used for blocking. Cells were then incubated with the primary antibody in blocking buffer, washed with PBS followed by an anti-

species secondary antibody, in the same blocking buffer. After washing, cells were re-blocked prior incubation with the second primary antibody, followed by an anti-species second secondary antibody, followed by DAPI (Invitrogen) staining and mounting in PermaFluor (Epredia). The primary antibodies used were human mAb anti-spike (clone VA14_R37, 25µg/ml) (10), rabbit anti-human C3d (1:200; Dako), and rabbit anti-human C5b-9 (1:200; abcam) all for 1h at RT, and rabbit anti-human CL-11 (1:100; Abbexa) 18h at 4°C. The secondary antibodies used were rat anti-human AlexaFluor-647 (1:200; Biolegend) and goat anti-rabbit FITC (1:200; Jackson Immunoresearch) for 1h at RT. Cells were imaged on a Nikon A1 inverted confocal (Eclipse Ti-E) microscope, using a Nikon Plan Apo lambda 60x/1.40 oil immersion lens, scanning at 1024 by 1024 pixels, resulting in a pixel size of 0.1 um/px. Images were acquired with a Galvano scanner controlled with NIS elements software. An average of 5 images were collected blind for each sample. All images were processed using Image J software (NIH) and quantitated as mean fluorescence intensity by circling the cell surface spike$^+$ or spike$^-$ cells as previously described (11).

Experimental primary human respiratory epithelial cell culture model.

Donor details.

Donor 1: female, 62 years, Caucasian

Donor 2: male, 71 years, Caucasian

Donor 3: male, 62 years, Caucasian

Cryopreserved human bronchial epithelial cells (HBECs) from donors with no history of lung disease were obtained from Epithelix Sàrl, Geneva, Switzerland (donor 1 and 3) and from PromoCell, Heidelberg, Germany (donor 2). Cells were expanded and seeded submerged at 2.12 x 10^5 cells/cm^2 in bronchial epithelial cell growth medium (BEGM, PromoCell) onto collagen coated (30 µg/ml) porous transwells (0.4 µm) until confluent. Apical media was then removed and replaced with basal differentiation medium (BEGM supplemented with non-essential amino acids,

sodium pyruvate, BSA, L-glutamine, penicillin, streptomycin and retinoic acid). Basal medium was replaced bi-weekly until beating cilia and mucus were observed (generally 4 weeks).

Virus infection of HBEC cultures. Prior to infection, the apical surface of the HBEC Air-Liquid Interface (ALI) culture was rinsed 2x with Hanks media, aspirated and incubated for 1.5 hr at 37°C and 5% CO_2 with mixtures of SARS-CoV-2 England 02/2020 (equivalent to an MOI of 0.1) that had been preincubated for 1 hr at 37°C with rCl-11 at either 0, 1.0 or 10.0 μg/ml (final concentration, as described above). The virus/rCl-11 mixture was then aspirated, the cells washed 3x with 200μl Hanks media and reincubated for 96 hr at 37°C. The last wash was collected for the 0 time point for virus assay. After 96 hr, 200μl Hanks media was applied ot the apical surface, incubated 15 min at 37°C, collected and titered by plaque assay.

Whole-mount immunostaining and imaging. SARS-CoV-2 infected and mock infected HBEC ALI cultures were washed 2x in PBS, fixed in 4% (w/v) paraformaldehyde in PBS for 20 min at room temperature, then re-washed 2x in PBS. ALI membranes were then cut from transwells prior to paraffin embedding and sectioning at 4μm thickness. For immunofluorescence staining, slides were subjected to devaxing (by standard procedures) and heat-induced antigen retrieval in 1 mM EDTA, pH 8.0 prior to blocking 1 hr in PBS containing 2% (w/v) BSA. Cultures were stained with primary antibodies specific for SARS-CoV-2 spike protein and CL-11 overnight at 4°C followed by incubation (1 hr) with flurochrome-conjugated species-specific secondary antibodies (**Table S2**) prepared in blocking buffer. Nuclei were visualised with DAPI (Invitrogen). Images were acquired on a Olympus BX51 microscope and processed using Image J software (NIH) to quantitate as mean fluorescence intensity.

Supporting Information Figures

A

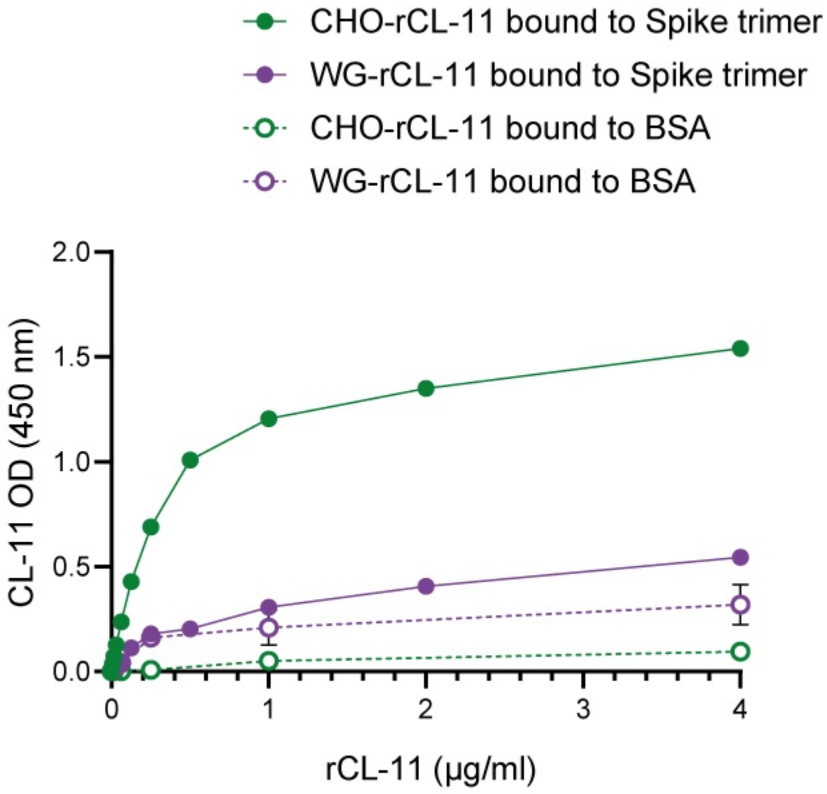
CHO-rCL-11 bound to Spike trimer
WG-rCL-11 bound to Spike trimer
CHO-rCL-11 bound to BSA
WG-rCL-11 bound to BSA
CL-11 OD (450 nm)
2.0
1.5
1.0
0.5
0.0
0
1
2
3
4
rCL-11 (µg/ml)

B

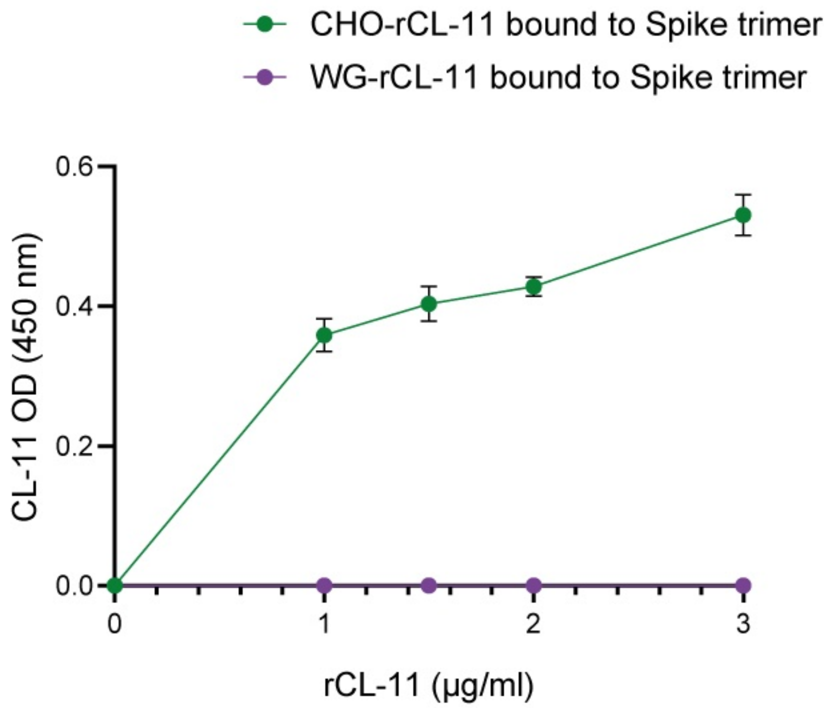
CHO-rCL-11 bound to Spike trimer
WG-rCL-11 bound to Spike trimer
CL-11 OD (450 nm)
0.6
0.4
0.2
0.0
0
1
2
3
rCL-11 (µg/ml)

Fig. S1. CHO-expressed but not wheat-germ-expressed rCL-11 binds to immobilised recombinant His tagged SARS-CoV-2 spike trimer protein. (**A**). A fixed concentration of His-tagged SARS-CoV-2 spike trimer (2 μg /ml) or BSA (2 μg /ml) as a control was immobilised on Maxisorp ELISA plates and tested for capture of CHO-expressed recombinant CL-11 and wheat-germ (WG) expressed rCL-11 over a concentration range (0- 4 μg /ml) of each respective protein. Bound proteins were detected with rabbit anti-CL-11 antibody, followed by goat anti-rabbit HRP and TMB enzyme substrate. Binding measured by absorbance at 450 nm.

(**B**). Nickel (high-binding ELISA plates) coated with His-tagged SARS-CoV-2 spike trimer (2 μg /ml) or BSA (2 μg /ml) as a control and tested for binding of CHO-expressed recombinant CL-11 and wheat-germ expressed rCL-11 according to the protocol described by Stravalaci et al (2022). Data (a, b) are enumerated by subtraction of values from blank wells from those recorded from sample wells and represent the mean ± S.E.M of n= 3 experiments performed in triplicate wells.

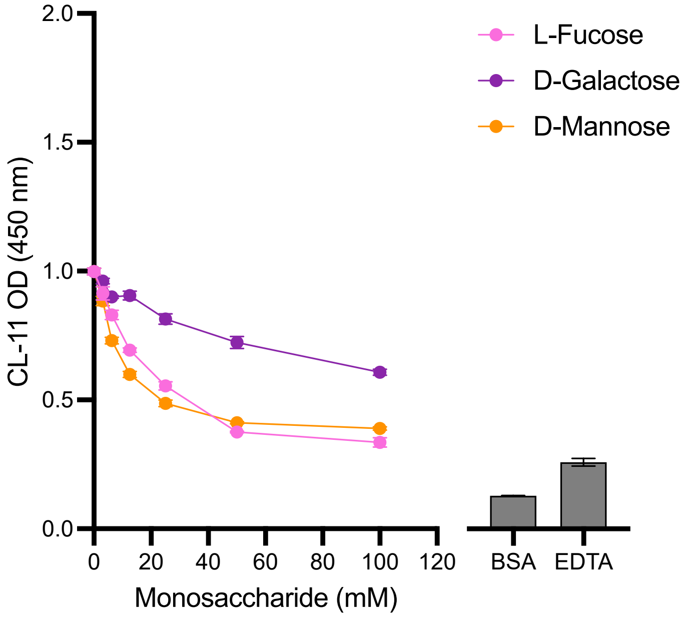

Fig. S2. SARS-CoV-2 interaction with the carbohydrate-binding domain of CL-11 is blocked by L-fucose, D-galactose and D-mannose. ELISA data show sugar specificity and Ca^{2+} binding-dependence of CL-11 bound to immobilised Wuhan spike trimer (2µg/ml), where rCL-11 (3µg/ml) had been pre-treated with L-fucose, D-mannose or D-galactose (0-100 mM) to block carbohydrate recognition of CL-11, or with EDTA (10 mM).

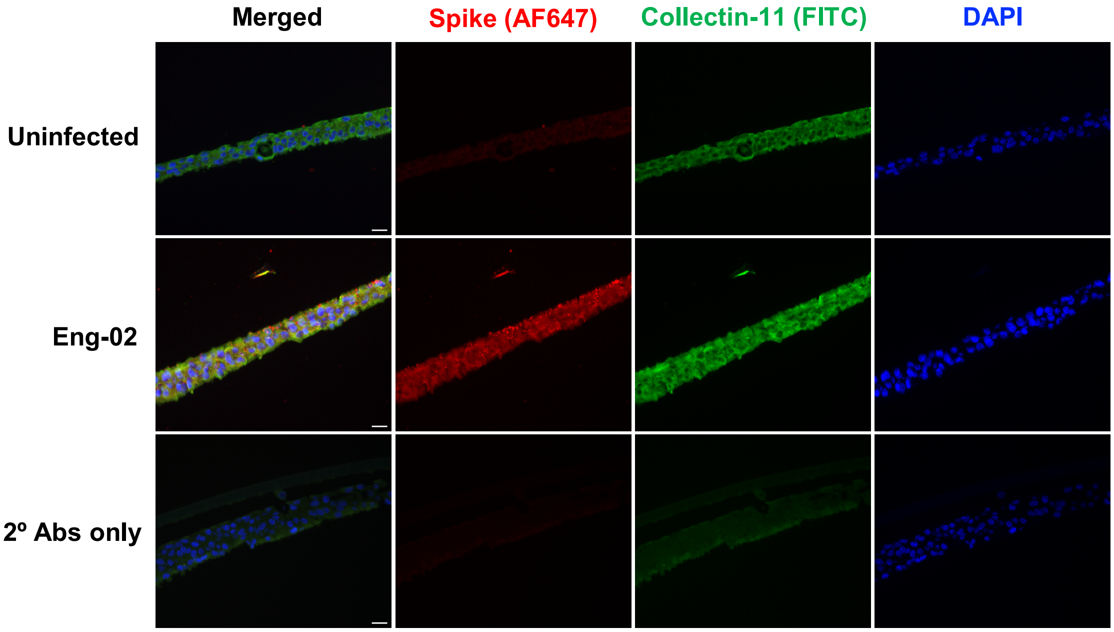

Fig. S3. SARS-CoV-2 infected primary human airway epithelial cells respond to cell intrinsic CL-11 activation. (**A, B, C)** Representative cross-section immunofluorescence images of primary human bronchial epithelial cells (HBECs) cultured at the air-liquid interface; control uninfected and virus infected with SARS-CoV-2 (Eng/02/2020) at MOI 0.1 at 96 hr post infection. Non-permeabilised cells were stained for cell surface SARS CoV-2 spike protein (red), collectin-11 (green) and DAPI (blue). Original magnification: x200; scale bars: 100 µm.

GATING STRATEGY

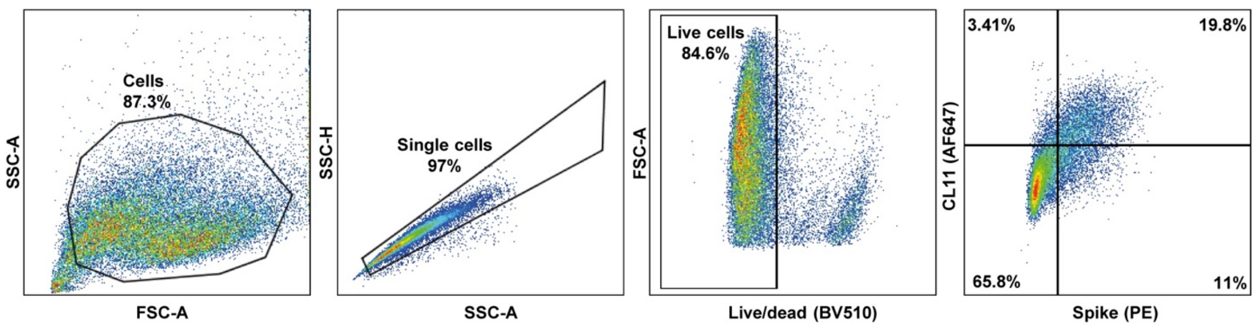

Fig. S4. Gating strategy used to identify CL-11 on the surface of spike transfected HEK293T cells. Spike-transfected or empty vector transfected HEK293T cells were gated based on FSC-A and SSC-A. Doublets were excluded based on the SSC-A and FSC-A gate. Live cells were selected based on the live-dead (BV510) gate and were further examined for cell surface CL-11 and spike protein.

Table S1. Data collection and refinement statistics.

Data collection	
Beamline	Diamond Light Source I04
Wavelength, *Å*	0.9759
Space group	$P\ 1\ 2_1\ 1$
a, b, c, Å	28.1, 105.8, 45.3
α, β, γ, °	90, 93.9, 90
Resolution, *Å*	41.57-1.80 (1.86 – 1.80)
No. reflections	23967 (2213)
R*sym*	0.048 (0.350)
CC(1/2)	0.997 (0.872)
I/δI	12.8 (2.4)
Completeness	98.0 (89.7)
Redundancy	3.1 (2.0)
Refinement	
Resolution, *Å*	41.57-1.80 (1.86 – 1.80)
No. reflections	23967 (2213)
Multiplicity	3.1 (2.0)
R*work*/R*free*	15.2/19.3
No. atoms	2249
Protein	1982
Ligands	39
Water	228
B-factors, $Å^2$	28.9
Protein	28.0
Ligand	33.4
Water	35.9
RMS (bonds) Å	0.014
RMS (angles) °	1.29

Statistics for the highest-resolution shell are shown in parentheses.

Table S2: Key antibodies used

Antibody	Clone	Fluorochrome/ Conjugate	Source	Identifier	Application
Rabbit anti-human CL-11	Polyclonal	-	Abbexa	abx003772	Flow Cytometry ICC/Confocal
Rabbit anti-human CL-11	Polyclonal	-	Abcam	ab238585	ELISA
Mouse anti-human C3d	7C10	-	Abcam	ab17453	ELISA
Rabbit anti-human C3d	Polyclonal	-	Dako/Agilent	A006302-2	ICC/Confocal/IF
Mouse anti-human C5b-9	aE11 (neoepitope)	-	Dako/Agilent	M077701-8	ELISA
Rabbit anti-human C5b-9	Polyclonal	-	Abcam	ab55811	ICC/Confocal
Human Anti-SARS-CoV-2 RBD	P008_108	-	Doores lab	P008_108	Flow Cytometry
Human Anti-SARS-CoV-2 RBD	VA14_R37	-	Doores lab	VA14_R37	ICC/Confocal/IF
Rat anti-human IgG Fc	M1310G05	PE	Biolegend	410708	Flow cytometry
Rat anti-human IgG Fc	M1310G05	AF647	Biolegend	410714	ICC/Confocal/IF
Goat anti-mouse IgG (H+L)	Polyclonal	HRP	Jackson ImmunoResearch	115-035-003	ELISA
Goat anti-rabbit IgG (H+L)	Polyclonal	FITC	Jackson ImmunoResearch	111-095-144	ICC/Confocal
Goat anti-rabbit IgG (H+L)	Polyclonal	HRP	Cell signaling	7074S	ELISA
Goat anti-rabbit IgG	Polyclonal	HRP	Cayman Chemical	10004301	ELISA

Supporting Information References

1. U. Venkatraman Girija *et al.*, Molecular basis of sugar recognition by collectin-K1 and the effects of mutations associated with 3MC syndrome. *BMC Biol* **13**, 27 (2015).
2. M. Stravalaci *et al.*, Recognition and inhibition of SARS-CoV-2 by humoral innate immunity pattern recognition molecules. *Nat Immunol* **23**, 275-286 (2022).
3. H. Khan *et al.*, TMPRSS2 promotes SARS-CoV-2 evasion from NCOA7-mediated restriction. *PLoS Pathog* **17**, e1009820 (2021).
4. C. Graham *et al.*, Neutralization potency of monoclonal antibodies recognizing dominant and subdominant epitopes on SARS-CoV-2 Spike is impacted by the B.1.1.7 variant. *Immunity* **54**, 1276-1289.e1276 (2021).

5. A. J. McCoy *et al.*, Phaser crystallographic software. *J Appl Crystallogr* **40**, 658-674 (2007).
6. P. Emsley, K. Cowtan, Coot: model-building tools for molecular graphics. *Acta Crystallogr D Biol Crystallogr* **60**, 2126-2132 (2004).
7. G. N. Murshudov, A. A. Vagin, E. J. Dodson, Refinement of macromolecular structures by the maximum-likelihood method. *Acta Crystallogr D Biol Crystallogr* **53**, 240-255 (1997).
8. Anonymous, The CCP4 suite: programs for protein crystallography. *Acta Crystallogr D Biol Crystallogr* **50**, 760-763 (1994).
9. P. D. Adams *et al.*, PHENIX: a comprehensive Python-based system for macromolecular structure solution. *Acta Crystallogr D Biol Crystallogr* **66**, 213-221 (2010).
10. J. Seow *et al.*, ChAdOx1 nCoV-19 vaccine elicits monoclonal antibodies with cross-neutralizing activity against SARS-CoV-2 viral variants. *Cell Rep* **39**, 110757 (2022).
11. M. H. Shihan, S. G. Novo, S. J. Le Marchand, Y. Wang, M. K. Duncan, A simple method for quantitating confocal fluorescent images. *Biochem Biophys Rep* **25**, 100916 (2021).
